# Supplementary material for: Local amphotericin B therapy for Cutaneous Leishmaniasis: A systematic review
Source: PLoS Negl Trop Dis. 2024 Apr 16;18(4):e0012127. doi: 10.1371/journal.pntd.0012127 (PMC11051593; doi:10.1371/journal.pntd.0012127)
Supplement: S2 Table — (DOCX) [file pntd.0012127.s002.docx]

| **MEDLINE**/Pubmed (n=1.952) | ((((((((((((((Leishmaniasis[MeSH Terms]) OR (Leishmaniasis[Title/Abstract])) OR ("Leishmania Infection"[Title/Abstract])) OR (Leishmaniases[Title/Abstract])) OR ("Infection, Leishmania"[Title/Abstract])) OR ("Infections, Leishmania"[Title/Abstract])) OR ("Leishmania Infections"[Title/Abstract])) OR ("Leishmaniasis, Cutaneous"[MeSH Terms])) OR (Leishmaniasis, Cutaneous[Title/Abstract])) OR ("Cutaneous Leishmaniasis"[Title/Abstract])) OR ("Cutaneous Leishmaniases"[Title/Abstract])) OR ("Leishmania braziliensis"[MeSH Terms])) OR ("Leishmania braziliensis"[Title/Abstract])) OR ("Leishmania (Viannia) braziliensis"[Title/Abstract])) AND ((((((("Amphotericin B"[MeSH Terms]) OR ("Amphotericin B"[Title/Abstract])) OR (Amphotericin[Title/Abstract])) OR (Fungizone[Title/Abstract])) OR ("Amphotericin B Cholesterol Dispersion"[Title/Abstract])) OR ("Amphotericin B Colloidal Dispersion"[Title/Abstract])) OR ("Amphocil"[Title/Abstract])) |
| --- | --- |
| **LILACS**/Virtual Health Library  (n=253) | ((mh:(Leishmaniose )) OR (Leishmaniose ) OR (mh:(Leishmaniasis )) OR (Leishmaniasis ) OR ("Infecção por Leishmania") OR ("Leishmania Infection") OR (mh:("Leishmaniose Cutânea")) OR ("Leishmaniose Cutânea") OR (mh:("Leishmaniasis, Cutaneous")) OR ("Leishmaniasis, Cutaneous") OR (mh:("Leishmaniasis Cutánea")) OR ("Leishmaniasis Cutánea") OR ("Leishmaniose Tegumentar Americana") OR ("Leishmaniose Americana") OR ("Leishmaniose do Novo Mundo") OR ("Leishmaniose do Velho Mundo") OR ("Úlcera de Bauru") OR (mh:("Leishmania braziliensis")) OR ("Leishmania braziliensis") OR ("Leishmania (Viannia) braziliensis") OR ("Leishmania braziliensis braziliensis")) AND ((mh:("Anfotericina B")) OR ("Anfotericina B") OR (mh:( "Amphotericin B")) OR ("Amphotericin B") OR (Amphotericin) OR (Fungizone) OR ("Amphotericin B Cholesterol Dispersion") OR ("Amphotericin B Colloidal Dispersion") OR (Amphocil)) |
| **Embase**  (n=4.092) | ('leishmaniasis'/exp OR leishmaniasis:ab,ti OR 'infection by leishmania':ab,ti OR 'infection of leishmania':ab,ti OR 'leishmania infection':ab,ti OR 'leishmanial infection':ab,ti OR 'leishmaniases':ab,ti OR 'leishmaniosis':ab,ti OR 'cutaneous leishmaniasis'/exp OR 'cutaneous leishmaniasis':ab,ti OR 'cutaneous leishmaniosis':ab,ti OR 'dermal leishmaniasis':ab,ti OR 'skin leishmaniasis':ab,ti OR 'leishmaniasis, skin':ab,ti OR 'leishmania braziliensis'/exp OR 'leishmania braziliensis':ab,ti OR leishmania:ab,ti OR viannia:ab,ti OR braziliensis:ab,ti OR 'leishmania braziliensis braziliensis':ab,ti) AND ('amphotericin b'/exp OR 'amphotericin b':ab,ti OR ambiosome:ab,ti OR 'amfotericin b':ab,ti OR amphocin:ab,ti OR amphotericin:ab,ti OR fungizone:ab,ti OR 'amphotericin b cholesterol dispersion':ab,ti OR 'amphotericin b colloidal dispersion':ab,ti OR 'amphocil':ab,ti) |
| **Cochrane Library**  (n=25) | 'Population ( "Leishmaniasis" OR "Cutaneous Leishmaniasis" OR "American Cutaneous/mucocutaneous Leishmaniasis" OR "Old World Cutaneous Leishmaniasis" ) OR Intervention "Agents Against Leishmaniasis And Trypanosomiasis" AND "Amphotericin B" OR "Amphotericin" OR "Amphotericin B Liposome"' |
| **Web of Science**  (n=2.261) | (Leishmaniasis or "Leishmaniasis, Cutaneous" or "Cutaneous Leishmaniasis" or "Leishmania braziliensis" or "Leishmania (Viannia) braziliensis") and ("Amphotericin B" or Amphotericin or fungizone or "Amphotericin B Cholesterol Dispersion" or "Amphotericin B Colloidal Dispersion" or amphoraic) |

S2 Table. **Search strategies**
