# Supplementary material for: Local amphotericin B therapy for Cutaneous Leishmaniasis: A systematic review
Source: PLoS Negl Trop Dis. 2024 Apr 16;18(4):e0012127. doi: 10.1371/journal.pntd.0012127 (PMC11051593; doi:10.1371/journal.pntd.0012127)
Supplement: S3 Table — (DOCX) [file pntd.0012127.s003.docx]

**List of excluded references**

| 1. FURTADO TA. [Treatment of cutaneous leishmaniasis americana by amphotericin B]. Hospital (Rio J). 1961 May;59:969-1001. Portuguese. PMID: 13702695. |
| --- |
| 2. MIRANDA JL, dos LIMA NS, da CUNHA J. [Amphotericin B in the treatment of American tegumental leishmaniasis]. Hospital (Rio J). 1961 Jun;59:1065-87. Portuguese. PMID: 13771161. |
| 3. Ganor S. The treatment of leishmaniasis recidivans with local injections of amphotericin B. Dermatol Int. 1967 Jul-Sep;6(3):141-3. doi: 10.1111/j.1365-4362.1967.tb05251.x. PMID: 5590113. |
| 4. Yesudian P, Thambiah AS. Amphotericin B therapy in dermal leishmanoid. Arch Dermatol. 1974 May;109(5):720-2. PMID: 4828540. |
| 5. Liubimov LK, Fateeva LI, Sergeeva LA. Eksperimental'naia kharakteristika protivoleĭshmanioznogo deĭstviia amfoteritsina B [Experimental characteristics of the antileishmaniasis action of amphotericin B]. Antibiotiki. 1979 Apr;24(4):281-4. Russian. PMID: 375822. |
| 6. Chevrel, B. Treatment of leishmaniosis by amphotericine B. Actualités Parasitologiques. Méd. Chir. Dig. 1984 - n° 13. pag. 463-464. |
| 7. Panosian CB, Barza M, Szoka F, Wyler DJ. Treatment of experimental cutaneous leishmaniasis with liposome-intercalated amphotericin B. Antimicrob Agents Chemother. 1984 May;25(5):655-6. doi: 10.1128/AAC.25.5.655. PMID: 6732232; |
| PMCID: PMC185609. |
| 8. Herbrecht R, Letscher V. Safety and efficacy of Intralipid emulsions of amphotericin B. J Antimicrob Chemother. 1997 Jul;40(1):137-9. doi:10.1093/jac/40.1.137. PMID: 9249219. |
| 9. Frankenburg S, Glick D, Klaus S, Barenholz Y. Efficacious topical treatment for murine cutaneous leishmaniasis with ethanolic formulations of amphotericin B. Antimicrob Agents Chemother. 1998 Dec;42(12):3092-6. doi: 10.1128/AAC.42.12.3092. PMID: 9835496; PMCID: PMC106004. |
| 10. Vardy D, Barenholz Y, Cohen R, Zvulunov A, Biton A, Klaus S, Frankenburg S. Topical amphotericin B for cutaneous leishmaniasis. Arch Dermatol. 1999 Jul;135(7):856-7. doi: 10.1001/archderm.135.7.856. PMID: 10411171. |
| 11. Plotnick AN. Lipid-based formulations of amphotericin B. J Am Vet Med Assoc. 2000 Mar 15;216(6):838-41. doi: 10.2460/javma.2000.216.838. PMID: 22570893. |
| 12. Vardy D, Barenholz Y, Naftoliev N, Klaus S, Gilead L, Frankenburg S. Efficacious topical treatment for human cutaneous leishmaniasis with ethanolic lipid amphotericin B. Trans R Soc Trop Med Hyg. 2001 Mar-Apr;95(2):184-6. doi:10.1016/s0035-9203(01)90158-0. PMID: 11355557. |
| 13. Zvulunov A, Cagnano E, Frankenburg S, Barenholz Y, Vardy D. Topical treatment of persistent cutaneous leishmaniasis with ethanolic lipid amphotericin B. Pediatr Infect Dis J. 2003 Jun;22(6):567-9. PMID: 12828157. |
| 14. [Silva, Marcos V](https://pesquisa.bvsalud.org/portal/?lang=pt&q=au:%22Silva,%20Marcos%20V%22); [Stocco, Joyce M](https://pesquisa.bvsalud.org/portal/?lang=pt&q=au:%22Stocco,%20Joyce%20M%22); [Rodrigues, Camila](https://pesquisa.bvsalud.org/portal/?lang=pt&q=au:%22Rodrigues,%20Camila%22); [Marques, Paula](https://pesquisa.bvsalud.org/portal/?lang=pt&q=au:%22Marques,%20Paula%22); [Baez, R. M](https://pesquisa.bvsalud.org/portal/?lang=pt&q=au:%22Baez,%20R.%20M%22); [Lindoso, José, A](https://pesquisa.bvsalud.org/portal/?lang=pt&q=au:%22Lindoso,%20Jos%C3%A9,%20A%22). Leishmaniose tegumentar americana (lta), tratamento alternativo com anfotericina b lipossomal / American tegumentary leishamaniasis (ATL), alternative treatment liposomal amphotericin B. [Rev. Soc. Bras. Med. Trop](http://portal.revistas.bvs.br/transf.php?xsl=xsl/titles.xsl&xml=http://catserver.bireme.br/cgi-bin/wxis1660.exe/?IsisScript=../cgi-bin/catrevistas/catrevistas.xis\|database_name=TITLES\|list_type=title\|cat_name=ALL\|from=1\|count=50&lang=pt&comefrom=home&home=false&task=show_magazines&request_made_adv_search=false&lang=pt&show_adv_search=false&help_file=/help_pt.htm&connector=ET&search_exp=Rev.%20Soc.%20Bras.%20Med.%20Trop); 36(supl. 1): 309-309, mar. 2003. |
| 15. Rosenthal, E. Amphotericin B lipid complex: A viewpoint. 2004. |
| 16. Richardson M. AmBisome today: expanding the perspectives on liposomal amphotericin B. Acta Biomed. 2006;77 Suppl 2:3-4. PMID: 16918058. |
| 17. Monzote L, Montalvo AM, Scull R, Miranda M, Abreu J. Combined effect of the essential oil from Chenopodium ambrosioides and antileishmanial drugs on promastigotes of Leishmania amazonensis. Rev Inst Med Trop Sao Paulo. 2007 Jul-Aug;49(4):257-60. doi: 10.1590/s0036-46652007000400012. PMID: 17823757. |
| 18. Lindoso, J A L. Resposta terapêutica de leishmaniose cutâneo-mucosa à anfotericina B lipossomal TT - Therapeutic response of mucocutaneous leishmaniasis with liposomal amphotericin B. 2009. |
| 19. del Rosal Rabes T, Baquero-Artigao F, Gómez Fernández C, García Miguel MJ, de Lucas Laguna R. Tratamiento de la leishmaniasis cutánea con anfotericina B liposomal [Treatment of cutaneous leishmaniasis with liposomal amphotericin B]. An Pediatr (Barc). 2010 Aug;73(2):101-2. Spanish. doi:10.1016/j.anpedi.2010.05.014. Epub 2010 Jul 7. PMID: 20615769. |
| 20. Rahimi-Moghaddam P, Ebrahimi SA, Ourmazdi H, Selseleh M, Karjalian M, Haj- Hassani G, Alimohammadian MH, Mahmoudian M, Shafiei M. In vitro and in vivo activities of Peganum harmala extract against Leishmania major. J Res Med Sci. 2011 Aug;16(8):1032-9. PMID: 22279479; PMCID: PMC3263080. |
| 21. Morizot G, Kendjo E, Mouri O, Thellier M, Pérignon A, Foulet F, Cordoliani F, Bourrat E, Laffitte E, Alcaraz I, Bodak N, Ravel C, Vray M, Grogl M, Mazier D, Caumes E, Lachaud L, Buffet PA; Cutaneous Leishmaniasis French Study Group. Travelers with cutaneous leishmaniasis cured without systemic therapy. Clin Infect Dis. 2013 Aug;57(3):370-80. doi: 10.1093/cid/cit269. Epub 2013 Apr 30. PMID: 23633111. |
| 22. [Abeer H. A. Mohamed-Ahmed](https://pubs.rsc.org/en/results?searchtext=Author%3AAbeer%20H.%20A.%20Mohamed-Ahmed),  [Karolina A. Les](https://pubs.rsc.org/en/results?searchtext=Author%3AKarolina%20A.%20Les),  [Simon L. Croft](https://pubs.rsc.org/en/results?searchtext=Author%3ASimon%20L.%20Croft),  [Stephen Brocchini](https://pubs.rsc.org/en/results?searchtext=Author%3AStephen%20Brocchini). Preparation and characterisation of amphotericin B-copolymer complex for the treatment of leishmaniasis. Polymer chemistry. 2013. |
| 23. Daftarian PM, Stone GW, Kovalski L, Kumar M, Vosoughi A, Urbieta M, Blackwelder P, Dikici E, Serafini P, Duffort S, Boodoo R, Rodríguez-Cortés A, Lemmon V, Deo S, Alberola J, Perez VL, Daunert S, Ager AL. A targeted and adjuvanted nanocarrier lowers the effective dose of liposomal amphotericin B and enhances adaptive immunity in murine cutaneous leishmaniasis. J Infect Dis. 2013 Dec 1;208(11):1914-22. doi: 10.1093/infdis/jit378. Epub 2013 Jul 29. PMID: 23901083; PMCID: PMC3814840. |
| 24. Mohamed-Ahmed AH, Seifert K, Yardley V, Burrell-Saward H, Brocchini S, Croft SL. Antileishmanial activity, uptake, and biodistribution of an amphotericin B and poly(α-Glutamic Acid) complex. Antimicrob Agents Chemother. 2013 Oct;57(10):4608-14. doi: 10.1128/AAC.02343-12. Epub 2013 Jun 24. PMID: 23796924; PMCID: PMC3811429 |
| 25. Solomon M, Schwartz E, Pavlotsky F, Sakka N, Barzilai A, Greenberger S. Leishmania tropica in children: a retrospective study. J Am Acad Dermatol. 2014 Aug;71(2):271-7. doi: 10.1016/j.jaad.2013.12.047. Epub 2014 Apr 26. PMID: 24775403. |
| 26. Ribeiro TG, Franca JR, Fuscaldi LL, Santos ML, Duarte MC, Lage PS, Martins VT, Costa LE, Fernandes SO, Cardoso VN, Castilho RO, Soto M, Tavares CA, Faraco A, Coelho EA, Chávez-Fumagalli MA. An optimized nanoparticle delivery system based on chitosan and chondroitin sulfate molecules reduces the toxicity of amphotericin B and is effective in treating tegumentary leishmaniasis. Int J Nanomedicine. 2014 Nov 19;9:5341-53. doi: 10.2147/IJN.S68966. PMID: 25429219; PMCID: PMC4242406. |
| 27. Henry, B. Worldwide diversity of leishmaniasis: Harmonized collection of data and isolates by the European “LeishMan” network: The LeishMan Network. 2015. |
| 28. Sbeghen MR, Voltarelli EM, Campois TG, Kimura E, Aristides SM, Hernandes L, Caetano W, Hioka N, Lonardoni MV, Silveira TG. Topical and Intradermal Efficacy of Photodynamic Therapy with Methylene Blue and Light-Emitting Diode in the Treatment of Cutaneous Leishmaniasis Caused by Leishmania braziliensis. J Lasers Med Sci. 2015 Summer;6(3):106-11. doi: 10.15171/jlms.2015.03. Epub 2015 Jun 28. PMID: 26464777; PMCID: PMC4599196. |
| 29. Mushtaq S, Dogra D, Dogra N. Clinical Response with intralesional Amphotericin B in the treatment of old world cutaneous leishmaniasis: a preliminary report. Dermatol Ther. 2016 Nov;29(6):398-405. doi:10.1111/dth.12377. Epub 2016 Aug 1. PMID: 27477764. |
| 30. Mendonça DV, Lage LM, Lage DP, Chávez-Fumagalli MA, Ludolf F, Roatt BM, Menezes-Souza D, Faraco AA, Castilho RO, Tavares CA, Barichello JM, Duarte MC, Coelho EA. Poloxamer 407 (Pluronic(®) F127)-based polymeric micelles for amphotericin B: In vitro biological activity, toxicity and in vivo therapeutic efficacy against murine tegumentary leishmaniasis. Exp Parasitol. 2016 Oct;169:34-42. doi: 10.1016/j.exppara.2016.07.005. Epub 2016 Jul 15. PMID: 27427166. |
| 31. Nikandish M, Goyonlo VM, Taheri AR, Kiafar B. Ocular Leishmaniasis Treated by Intralesional Amphotericin B. Middle East Afr J Ophthalmol. 2016 Jan-Mar;23(1):153-5. doi: 10.4103/0974-9233.171801. PMID: 26957858; PMCID: PMC4759897. |
| 32. Alcántara-Reifs CM, Garnacho-Saucedo G, Salido-Vallejo R, Vélez García-Nieto A. Topical Amphotericin B for the treatment of localized cutaneous leishmaniasis. Dermatol Ther. 2017 Jan;30(1). doi: 10.1111/dth.12402. Epub 2016 Sep 16. PMID: 27636906. |
| 33. Kaur L, Abhijeet, Jain SK. Safe and Effective Delivery of Amphotericin B: A Survey of Patents. Recent Pat Nanotechnol. 2017;11(3):214-234. doi: 10.2174/1872210511666170105130210. PMID: 28056751. |
| 34. Mehrizi TZ, Ardestani MS, Molla Hoseini MH, Khamesipour A, Mosaffa N, Ramezani A. Novel nano-sized chitosan amphotericin B formulation with considerable improvement against Leishmania major. Nanomedicine (Lond). 2018 Dec;13(24):3129-3147. doi: 10.2217/nnm-2018-0063. Epub 2018 Nov 22. PMID: 30463469. |
| 35. Wijnant GJ, Van Bocxlaer K, Yardley V, Harris A, Murdan S, Croft SL. Relation between Skin Pharmacokinetics and Efficacy in AmBisome Treatment of Murine Cutaneous Leishmaniasis. Antimicrob Agents Chemother. 2018 Feb 23;62(3):e02009-17. doi: 10.1128/AAC.02009-17. PMID: 29263075; PMCID: PMC5826151. |
| 36. Wijnant GJ, Van Bocxlaer K, Fortes Francisco A, Yardley V, Harris A, Alavijeh M, Murdan S, Croft SL. Local Skin Inflammation in Cutaneous Leishmaniasis as a Source of Variable Pharmacokinetics and Therapeutic Efficacy of Liposomal Amphotericin B. Antimicrob Agents Chemother. 2018 Sep 24;62(10):e00631-18. doi: 10.1128/AAC.00631-18. PMID: 30082295; PMCID: PMC6153808. |
| 37. Eskandari SE, Firooz A, Nassiri-Kashani M, Jaafari MR, Javadi A, Miramin Mohammadi A, Khamesipour A. Safety Evaluation of Topical Application of Nano-Liposomal Form of Amphotericin B (SinaAmpholeish) on Healthy Volunteers: Phase I Clinical Trial. Iran J Parasitol. 2019 Apr-Jun;14(2):197-203. PMID: 31543907; PMCID: PMC6737359. |
| 38. Gupta A, Sardana K, Ahuja A, Kishan Gautam R. Complete cure of a large complex cutaneous leishmaniasis with a nonethanolic lipid based-amphotericin B gel. Clin Exp Dermatol. 2019 Oct;44(7):807-810. doi: 10.1111/ced.13883. Epub 2018 Dec 28. PMID: 30592076. |
| 39. Zur E. Topical Treatment of Cutaneous Leishmaniasis in Israel, Part 1. Int J Pharm Compd. 2019 May-Jun;23(3):200-207. PMID: 31085788. |
| 40. Lanza JS, Pomel S, Loiseau PM, Frézard F. Recent advances in amphotericin B delivery strategies for the treatment of leishmaniases. Expert Opin Drug Deliv. 2019 Oct;16(10):1063-1079. doi: 10.1080/17425247.2019.1659243. Epub 2019 Aug 30. PMID: 31433678. |
| 41. [Momin Khan](https://ietresearch.onlinelibrary.wiley.com/authored-by/Khan/Momin), [Akhtar Nadhman](https://ietresearch.onlinelibrary.wiley.com/authored-by/Nadhman/Akhtar), [Walayat Shah](https://ietresearch.onlinelibrary.wiley.com/authored-by/Shah/Walayat), [Imran Khan](https://ietresearch.onlinelibrary.wiley.com/authored-by/Khan/Imran), [Masoom Yasinzai](https://ietresearch.onlinelibrary.wiley.com/authored-by/Yasinzai/Masoom). Formulation and characterisation of a self-nanoemulsifying drug delivery system of amphotericin B for the treatment of leishmaniasis. [IET Nanobiotechnology](https://ietresearch.onlinelibrary.wiley.com/journal/1751875x) [Volume 13, Issue 5](https://ietresearch.onlinelibrary.wiley.com/toc/1751875x/2019/13/5) p. 477-483. 2019. |
| 42. Zadeh Mehrizi T, Khamesipour A, Shafiee Ardestani M, Ebrahimi Shahmabadi H, Haji Molla Hoseini M, Mosaffa N, Ramezani A. Comparative analysis between four model nanoformulations of amphotericin B-chitosan, amphotericin B-dendrimer, betulinic acid-chitosan and betulinic acid-dendrimer for treatment of <i>Leishmania major</i>: real-time PCR assay plus. Int J Nanomedicine. 2019 Sep 24;14:7593-7607. doi: 10.2147/IJN.S220410. PMID: 31802863; PMCID: PMC6831986. |
| 43. de Souza RM, Maranhão RC, Tavares ER, Filippin-Monteiro FB, Nicodemo AC, Morikawa AT, Kanashiro EHY, Amato VS. Lipid nanoparticles for amphotericin delivery in the treatment of American tegumentary leishmaniasis. Drug Deliv Transl Res. 2020 Apr;10(2):403-412. doi: 10.1007/s13346-019-00677-4. PMID: 31701487. |
| 44. Parvez S, Yadagiri G, Singh A, Karole A, Singh OP, Sundar S, Mudavath SL. Improvising anti-leishmanial activity of amphotericin B and paromomycin using co-delivery in d-α-tocopheryl polyethylene glycol 1000 succinate (TPGS) tailored nano-lipid carrier system. Chem Phys Lipids. 2020 Sep;231:104946. doi: 10.1016/j.chemphyslip.2020.104946. Epub 2020 Jul 1. PMID: 32621810. |
| 45. Dos Santos Matos AP, Lopes DCDXP, Peixoto MLH, da Silva Cardoso V, Vermelho AB, Santos-Oliveira R, Viçosa AL, Holandino C, Ricci-Júnior E. Development, characterization, and anti-leishmanial activity of topical amphotericin B nanoemulsions. Drug Deliv Transl Res. 2020 Dec;10(6):1552-1570. doi:10.1007/s13346-020-00821-5. PMID: 32676952. |
| 46. Riezk A, Van Bocxlaer K, Yardley V, Murdan S, Croft SL. Activity ofAmphotericin B-Loaded Chitosan Nanoparticles against Experimental Cutaneous Leishmaniasis. Molecules. 2020 Sep 2;25(17):4002. doi: 10.3390/molecules25174002. PMID: 32887341; PMCID: PMC7504813. |
| 47. Saqib M, Ali Bhatti AS, Ahmad NM, Ahmed N, Shahnaz G, Lebaz N, Elaissari A. Amphotericin B Loaded Polymeric Nanoparticles for Treatment of <i>Leishmania</i> Infections. Nanomaterials (Basel). 2020 Jun 12;10(6):1152. doi:10.3390/nano10061152. PMID: 32545473; PMCID: PMC7353296 |
| 48. Carvalheiro M, Vieira J, Faria-Silva C, Marto J, Simões S. Amphotericin B-loaded deformable lipid vesicles for topical treatment of cutaneous leishmaniasis skin lesions. Drug Deliv Transl Res. 2021 Apr;11(2):717-728. doi:10.1007/s13346-021-00910-z. Epub 2021 Feb 3. PMID: 33534106. |
| 49. Silva-Carvalho R, Fidalgo J, Melo KR, Queiroz MF, Leal S, Rocha HA, Cruz T, Parpot P, Tomás AM, Gama M. Development of dextrin-amphotericin B formulations for the treatment of Leishmaniasis. Int J Biol Macromol. 2020 Jun 15;153:276-288. doi: 10.1016/j.ijbiomac.2020.03.019. Epub 2020 Mar 5. Erratum in: Int J Biol Macromol. 2021 Jan 1;166:1619. PMID: 32145228. |
| 50. Eskandari SE, Khamesipour A, Jaafari MR, Javadi A, Mohammadi AM, Valian HK, Nassiri-Kashani M, Goyonlo VM, Firooz A. Combination of topical liposomal amphotericin B and Glucantime in comparison with glucantime alone for the treatment of anthroponotic cutaneous leishmaniasis (ACL) caused by <i>Leishmania tropica</i>: study protocol for a randomized, controlled trial. Iran J Microbiol. 2021 Oct;13(5):718-723. doi: 10.18502/ijm.v13i5.7440. PMID: 34900170; PMCID: PMC8629814. |
| 51. Diociaiuti A, Giancristoforo S, Calò Carducci FI, Bracaglia C, Boni A, Pane S, Onetti Muda A, De Benedetti F, Putignani L, El Hachem M. Auricular leishmaniasis in a child successfully treated with intralesional amphotericin B. Pediatr Dermatol. 2022 Sep;39(5):832-833. doi: 10.1111/pde.15046. Epub 2022 Jun 11. PMID: 35689474; PMCID: PMC9796609. |
| 52. Knapp C 3rd, Vaz L, Onoday H, Small A. Dual treatment of cutaneous leishmaniasis with topical amphotericin B and photodynamic therapy in a pediatric patient. Pediatr Dermatol. 2022 Sep;39(5):761-763. doi:10.1111/pde.15042. Epub 2022 May 30. PMID: 35636952 |
