# Supplementary material for: Local amphotericin B therapy for Cutaneous Leishmaniasis: A systematic review
Source: PLoS Negl Trop Dis. 2024 Apr 16;18(4):e0012127. doi: 10.1371/journal.pntd.0012127 (PMC11051593; doi:10.1371/journal.pntd.0012127)
Supplement: S4 Table — (DOCX) [file pntd.0012127.s004.docx]

**S4 Table.** Assessing the certainty of evidence using GRADE

| 1. **Should Amphotericin B topic be used for cutaneous leishmaniasis?** | | | | | | | | | | | |
| --- | --- | --- | --- | --- | --- | --- | --- | --- | --- | --- | --- |
| **Certainty assessment** | | | | | | | **Summary of findings** | | | | |
| **Participants (studies) Follow-up** | **Risk of bias** | **Inconsistency** | **Indirectness** | **Imprecision** | **Publication bias** | **Overall certainty of evidence** | **Study event rates (%)** | | **Relative effect (95% CI)** | **Anticipated absolute effects** | |
|  |  |  |  |  |  |  | **With coltrol** | **With amphotericin B** |  | **Risk control** | **Risk difference with amphotericin B** |
| **Cure** | | | | | | | | | | | |
| 152 (4 observational studies) | serious^a^ | very serious^b^ | not serious | very serious^c^ | none | ⨁◯◯◯ Very low | **-** | 65/162 (40.1%) | **-** | **-** | **-** |

**CI:** confidence interval; **OR:** odds ratio

#### Explanations

a. Important limitations for at least one domain

b. there is variability in the effect estimate and high heterogeneity

c. there is an inaccuracy in the real estimation of the effect, which may also be related to the small sample size

| 1. **Should Amphotericin B intralesional be used for cutaneous leishmaniasis?** | | | | | | | | | | | |
| --- | --- | --- | --- | --- | --- | --- | --- | --- | --- | --- | --- |
| **Certainty assessment** | | | | | | | **Summary of findings** | | | | |
| **Participants (studies) Follow-up** | **Risk of bias** | **Inconsistency** | **Indirectness** | **Imprecision** | **Publication bias** | **Overall certainty of evidence** | **Study event rates (%)** | | **Relative effect (95% CI)** | **Anticipated absolute effects** | |
|  |  |  |  |  |  |  | **With meglumine antimoniate** | **With amphotericin B** |  | **Risk with meglumine antimoniate** | **Risk difference with amphotericin B** |
| **Cure** | | | | | | | | | | | |
| 143 (3 observational studies) | serious^a^ | very serious^b^ | not serious | very serious^c^ | none | ⨁◯◯◯ Very low | **-** | 95/143 (66.4%) | **-** | **-** | **-** |

**CI:** confidence interval; **OR:** odds ratio

#### Explanations

a. Important limitations for at least one domain

b. there is variability in the effect estimate and high heterogeneity

c. there is an inaccuracy in the real estimation of the effect, which may also be related to the small sample size

| 1. **Should amphotericin B intralesional vs. meglumine antimoniate intralesional be used for cutaneous leishmaniasis ?** | | | | | | | | | | | |
| --- | --- | --- | --- | --- | --- | --- | --- | --- | --- | --- | --- |
| **Certainty assessment** | | | | | | | **Summary of findings** | | | | |
| **Participants (studies) Follow-up** | **Risk of bias** | **Inconsistency** | **Indirectness** | **Imprecision** | **Publication bias** | **Overall certainty of evidence** | **Study event rates (%)** | | **Relative effect (95% CI)** | **Anticipated absolute effects** | |
|  |  |  |  |  |  |  | **With meglumine antimoniate** | **With amphotericin B** |  | **Risk with meglumine antimoniate** | **Risk difference with amphotericin B** |
| **Cure** | | | | | | | | | | | |
| 162 (2 observational studies) | very serious^a^ | very serious^b^ | not serious | very serious^c^ | none | ⨁◯◯◯ Very low | 44/90 (48.9%) | 40/72 (55.6%) | **OR 1.78** (0.35 to 9.16) | 489 per 1.000 | **141 more per 1.000** (from 238 fewer to 409 more) |

**CI:** confidence interval; **OR:** odds ratio

#### Explanations

a. Important limitations for more than one domain

b. there is variability in the effect estimate and high heterogeneity.

c. there is an inaccuracy in the real estimation of the effect, which may also be related to the small sample size.
