# Supplementary material for: Local amphotericin B therapy for Cutaneous Leishmaniasis: A systematic review
Source: PLoS Negl Trop Dis. 2024 Apr 16;18(4):e0012127. doi: 10.1371/journal.pntd.0012127 (PMC11051593; doi:10.1371/journal.pntd.0012127)
Supplement: Attachment — Submitted filename: PNTD-D-23-00751_reviewer (first review).pdf [file pntd.0012127.s005.pdf]

PLOS Neglected Tropical Diseases

Local amphotericin B therapy for cutaneous leishmaniasis: a systematic review

--Manuscript Draft--

Manuscript Number:	PNTD-D-23-00751
Full Title:	Local amphotericin B therapy for cutaneous leishmaniasis: a systematic review
Short Title:	LOCAL AMPHOTERICIN B FOR CUTANEOUS LEISHMANIASIS
Article Type:	Research Article
Keywords:	Cutaneous leishmaniasis; Local treatment; Amphotericin B
Abstract:	Background: Cutaneous leishmaniasis (CL) is recognized for causing ulcers distributed throughout the body; such ulcers can be disfiguring and may intrinsically attract a social stigma. Local treatment using available drugs has been explored to reduce systemic absorption and the levels of toxicity currently observed with the available treatments. This review aimed to systematically gather data on the efficacy of amphotericin B (AmB) in local administration for the treatment of cutaneous leishmaniasis in preclinical or clinical studies. Methodology: Structured searches were performed in the main databases until October 30, 2022. For clinical studies, only those reporting cure rates were included, while for preclinical studies, any efficacy outcome was considered. Exclusion criteria were nonoriginal studies, in vitro studies, studies with fewer than 10 patients treated, and studies evaluating AmB combined with another active antileishmanial component (PROSPERO: CRD42021265854). Principal findings: In total, 21 studies were identified: 16 preclinical and five clinical studies. The global cure rate in the clinical studies was evaluated at 56.9% (CI: 41.1–71.4%), with great heterogeneity (I2=82,8) in the design, drug formulation, and route of administration of AmB. In summary, although this review confirms that local routes of administration of AmB are the object of clinical and preclinical investigation, our results confirm the lack of alignment between these two successive steps in the development of new therapeutic technologies, as well as the lack of standardization of minimum parameters and outcomes that allow direct comparison and pooling of different studies. Conclusions: The results gathered thus far suggest that AmB via local administration is a promising strategy that needs to be evaluated in well-designed studies, with the potential to compose combined LC treatment regimens, resulting in the expansion of therapeutic alternatives and possibly the reduction of toxicity involved with parenteral treatments.
Additional Information:	
Question	Response
**Government Employee**<br><br>Are you or any of the contributing authors an employee of the United States government?<br><br>Manuscripts authored by one or more US Government employees are not copyrighted, but are licensed under a CC0 Public Domain Dedication, which allows unlimited distribution and reuse of the article for any lawful purpose. This is a legal requirement for US Government employees.	No - No authors are employees of the U.S. government.

Powered by Editorial Manager® and ProduXion Manager® from Aries Systems Corporation

This will be typeset if the manuscript is accepted for publication.	
**Financial Disclosure**<br><br>Enter a financial disclosure statement that describes the sources of funding for the work included in this submission. Review the submission guidelines for detailed requirements. View published research articles from *PLOS Neglected Tropical Diseases* for specific examples.<br><br>This statement is required for submission and **will appear in the published article** if the submission is accepted. Please make sure it is accurate.<br><br>**Funded studies**<br>Enter a statement with the following details:<br>• Initials of the authors who received each award<br>• Grant numbers awarded to each author<br>• The full name of each funder<br>• URL of each funder website<br>• Did the sponsors or funders play any role in the study design, data collection and analysis, decision to publish, or preparation of the manuscript?<br><br>Did you receive funding for this work?	Yes
Please add funding details.<br>as follow-up to "**Financial Disclosure**<br><br>Enter a financial disclosure statement that describes the sources of funding for the work included in this submission. Review the submission guidelines for detailed requirements. View published research articles from *PLOS Neglected Tropical Diseases* for specific examples.<br><br>This statement is required for submission and **will appear in the published article** if the submission is accepted. Please make sure it is accurate.	LLA received grant number Finance Code 001 by the Coordenação de Aperfeiçoamento de Pessoal de Nível Superior (CAPES), https://www.capes.gov.br. MLF received grant number 151891/2022-2 by the Conselho Nacional de Desenvolvimento Científico e Tecnológico (CNPq), http://www.cnpq.br. GC received grant number 301384/2019-3 by the Conselho Nacional de Desenvolvimento Científico e Tecnológico (CNPq), http://www.cnpq.br. The funders had no role in study design, data collection and analysis, decision to<br>publish, or preparation of the manuscript.

Powered by Editorial Manager® and ProduXion Manager® from Aries Systems Corporation

**Funded studies**<br>Enter a statement with the following details:<br>• Initials of the authors who received each award<br>• Grant numbers awarded to each author<br>• The full name of each funder<br>• URL of each funder website<br>• Did the sponsors or funders play any role in the study design, data collection and analysis, decision to publish, or preparation of the manuscript?<br><br>Did you receive funding for this work?"	
Please select the country of your main research funder (please select carefully as in some cases this is used in fee calculation).<br>as follow-up to "**Financial Disclosure**<br><br>Enter a financial disclosure statement that describes the sources of funding for the work included in this submission. Review the submission guidelines for detailed requirements. View published research articles from *PLOS Neglected Tropical Diseases* for specific examples.<br><br>This statement is required for submission and **will appear in the published article** if the submission is accepted. Please make sure it is accurate.<br><br>**Funded studies**<br>Enter a statement with the following details:<br>• Initials of the authors who received each award<br>• Grant numbers awarded to each author<br>• The full name of each funder<br>• URL of each funder website<br>• Did the sponsors or funders play any role in the study design, data collection and analysis, decision to publish, or preparation of the manuscript?<br><br>Did you receive funding for this work?"	BRAZIL - BR
**Competing Interests**	The authors have declared that no competing interests exist.

Powered by Editorial Manager® and ProduXion Manager® from Aries Systems Corporation

On behalf of all authors, disclose any competing interests that could be perceived to bias this work.<br><br>This statement will be typeset if the manuscript is accepted for publication.<br><br>Review the instructions link below and PLOS NTDs' competing interests policy to determine what information must be disclosed at submission.	
**Data Availability**<br><br>Provide a **Data Availability Statement** in the box below. This statement should detail where the data used in this submission can be accessed. This statement will be typeset if the manuscript is accepted for publication.<br><br>Before publication, authors are required to make all data underlying their findings fully available, without restriction. Review our PLOS Data Policy page for detailed information on this policy. Instructions for writing your Data Availability statement can be accessed via the Instructions link below.	All relevant data are within the manuscript and its Supporting Information files.

Manuscript Click here to access/download;Manuscript;Manuscript.docx

LOCAL AMPHOTERICIN B THERAPY FOR CUTANEOUS LEISHMANIASIS: A SYSTEMATIC REVIEW

LOCAL AMPHOTERICIN B FOR CUTANEOUS LEISHMANIASIS

Líndicy Leidicy Alves*; Mariana Lourenço Freire; Isadora Lana Troian; Eliane de Morais-Teixeira; Gláucia Cota

Current Address:

Clinical Research and Public Policy Group on Infectious and Parasitic Diseases – René Rachou Institute - Fundação Oswaldo Cruz – FIOCRUZ, Belo Horizonte, Minas Gerais, Brazil.

*Corresponding Author: E-mail: lindicy.leidicy@hotmail.com

ABSTRACT

Background

Cutaneous leishmaniasis (CL) is recognized for causing ulcers distributed throughout the body; such ulcers can be disfiguring and may intrinsically attract a social stigma. Local treatment using available drugs has been explored to reduce systemic absorption and the levels of toxicity currently observed with the available treatments. This review aimed to systematically gather data on the efficacy of amphotericin B (AmB) in local administration for the treatment of cutaneous leishmaniasis in preclinical or clinical studies.

Methodology

Structured searches were performed in the main databases until October 30, 2022. For clinical studies, only those reporting cure rates were included, while for preclinical studies, any efficacy outcome was considered. Exclusion criteria were nonoriginal studies, in vitro studies, studies with fewer than 10 patients treated, and studies

evaluating AmB combined with another active antileishmanial component (PROSPERO: CRD42021265854).

Principal findings

In total, 21 studies were identified: 16 preclinical and five clinical studies. The global cure rate in the clinical studies was evaluated at 56.9% (CI: 41.1–71.4%), with great heterogeneity (I^2=82,8) in the design, drug formulation, and route of administration of AmB. In summary, although this review confirms that local routes of administration of AmB are the object of clinical and preclinical investigation, our results confirm the lack of alignment between these two successive steps in the development of new therapeutic technologies, as well as the lack of standardization of minimum parameters and outcomes that allow direct comparison and pooling of different studies.

Conclusions

The results gathered thus far suggest that AmB via local administration is a promising strategy that needs to be evaluated in well-designed studies, with the potential to compose combined LC treatment regimens, resulting in the expansion of therapeutic alternatives and possibly the reduction of toxicity involved with parenteral treatments.

Keywords: Cutaneous leishmaniasis; Local treatment; Amphotericin B.

Author summary

Cutaneous leishmaniasis (CL) is a neglected disease recognized for causing ulcers distributed throughout the body; such ulcers can be disfiguring and may intrinsically attract a social stigma. Furthermore, the currently available therapeutic arsenal is admittedly limited and associated with significant toxicity. Long therapeutic regimens

and parenteral administration have been considered in recent years as a strategy to be replaced. In this scenario, the search for new treatment alternatives for CL is considered a priority by the World Health Organization, which has been expanding its treatment recommendations with alternatives for local use. In this study, we performed an extensive literature search to gather evidence on the efficacy of amphotericin B (AmB) in local administration for the treatment of CL in preclinical or clinical studies. In total, 21 studies, 16 preclinical studies and five clinical studies were identified. The global cure rate for clinical studies addressing intralesional infiltration and topical application was estimated at 56.9% (CI: 41.1–71.4%). High heterogeneity in the design, groups and route of administration of AmB among studies was observed, requiring caution in interpreting and extrapolating of this pooled rate. In summary, the results show that local routes of administration of AmB are a promising strategy and need to be evaluated in studies with adequate design, enabling the expansion of therapeutic alternatives for the treatment of CL.

INTRODUCTION

Leishmaniasis is an infectious parasitic disease that occurs in tropical low-income countries, usually with limited access to health care. The clinical manifestation varies according to the species of *Leishmania,* immune response and, probably, other concomitant host conditions. Compared to the visceral and mucosal or mucocutaneous leishmaniasis forms (VL and MCL), cutaneous leishmaniasis (CL) is the most prevalent, with more than 220 thousand new cases worldwide [1,2].

CL is recognized for causing ulcers that can be disfiguring and distributed throughout the body but are unlikely to progress to a fatal outcome. The disease is intrinsically related to social stigma, even after treatment, due to the risk of permanent scarring and relapses [3,4]. The optimal treatment is not yet available for leishmaniasis, and meglumine antimoniate and amphotericin B (AmB), both of which are related to significant toxicity, are the most used drugs [5].

AmB is recognized for its high leishmanicidal effect. Initially, made available in a formulation using sodium deoxycholate as a solubilizing agent, this drug has also become known for its potential for nephrotoxicity [6]. With a focus on reducing the occurrence of this significant adverse event, lipid formulations of AmB were developed, including liposomal AmB (L-Amb), a presentation based on AmB incorporation into liposomes, which still traditionally requires intravenous administration [7,8].

Another strategy to overcome adverse events with the systemic use of AmB would be the local use of the drug, an approach already taken with antimony derivatives, either by infiltration into the lesion or topical application [9]. The local administration route could theoretically lead to high drug concentrations at the site of infection, which could reduce the total dose applied and, consequently, the occurrence of toxic effects. In addition, local drug administration would reduce the treatment complexity and costs [10].

This review aimed to systematically gather data on the therapeutic response with different formulations of AmB used locally in clinical and preclinical studies. The main

objective was to map the current level of evidence with the local approach to support a new therapeutic approach development plan and the design of future clinical trials.

METHODS

Protocol and registration

The protocol of this review was registered in the International Prospective Register of Systematic Reviews (PROSPERO: CRD42021265854). This systematic review was conducted according to the Preferred Reporting Items for Systematic Reviews and Meta-Analyses (PRISMA - S1 Table) [11].

Information sources and search strategy

A literature search was conducted to identify studies assessing the efficacy of AmB used locally for the treatment of cutaneous leishmaniasis lesions in preclinical and clinical studies. Structured searches were performed in MEDLINE (accessed by PubMed), Latin American and Caribbean Literature on Health Sciences (accessed by Virtual Health Library), Excerpta Medica Database (Embase), Cochrane Library and Web of Science. Search strategies combining the keywords related to cutaneous *leishmaniasis AND amphotericin* B were constructed for each database, and the strategies are summarized in the Supplementary Materials (S2 Table). A manual search was also performed by analysis of the reference lists of selected articles. All searches were performed up to October 30, 2022.

Selection criteria

Original clinical or preclinical studies reporting the efficacy of locally administered treatment based on any formulation of AmB for cutaneous leishmaniasis were included. For clinical studies, only those reporting the cure rates were included, whereas for preclinical studies, any other efficacy outcome was considered. Exclusion criteria were as follows: (I) nonoriginal studies, including literature reviews, editorials, brief communications, and case reports; (II) in vitro studies; (III) studies with fewer than 10 treated patients; and (IV) studies evaluating AmB combined with another antileishmanial active component. There were no language or publication date restrictions.

Selection process, data extraction and outcomes

For each database, all recovered articles were added to the Mendeley reference manager for duplicate citation exclusion. According to the inclusion and exclusion criteria, two independent reviewers (LLA and MLF) analyzed each publication by title and abstract using Rayyan software [12]. Disagreements were resolved by consensus or with a third reviewer (GC). Data were extracted from the included studies by two independent reviewers (LLA and MLF).

For preclinical studies, methodological characteristics of the included studies, such as animal models, *Leishmania* species, infection sites, treatment schedules, comparator groups, outcomes of interest and follow-up were extracted.

For clinical studies, standard data extraction forms were used to collect the main study and population characteristics and outcomes. For these studies, the outcome of interest was the initial cure rate. Considering D1 as the first day of treatment and using the definitions proposed by Olliaro (2013) [13], which establishes initial cure that evaluated in D90, here we arbitrarily assumed as the initial cure rate that assessed between D42 and D90. The cure rate was expressed as the number of cases cured by the total number of cases treated with a given intervention (intention-to-treat approach). Losses observed during follow-up were considered therapeutic failures.

To estimate the relapse rate, only patients considered previously cured were included. The safety of antileishmanial therapy was captured in each study as the number of adverse events per total number of evaluated patients (or treatments, if this was the only information available).

Data synthesis and statistical analysis

Comprehensive Meta-Analysis software v.3.0 was used to perform a one-group meta-analysis of study arms using a given treatment (pooled rates) based on an assessment of the baseline cure rate, as arbitrarily adopted (D90), reported in the original studies. Clinical cure rates were calculated according to the intention-to-treat approach, that means that the analysis was based on the total number of randomly assigned participants, irrespective of how the original study's authors analyzed the data. We used the inconsistency (I^2) statistic to evaluate heterogeneity.

Study quality assessment

The quality of evidence was evaluated by two independent researchers (LAA and EMT for preclinical studies and LAA and MLF for clinical studies) using the recommended tools according to the type of study, as follows: SYRCLE risk, an adapted version of the Cochrane RoB tool of bias, was used for animal studies [14], the Newcastle Ottawa Scale (NOS) for nonrandomized clinical trials [15], and the Cochrane risk of bias score (RoB 2), for randomized controlled trials (RCTs) [16].

RESULTS

Literature search

A total of 8,583 studies were recovered from MEDLINE (1,952), LILACS (253), Cochrane Library (25), Web of Science (2,261) and Embase (4,092). Initially, all duplicates were removed, and 4,911 titles and abstracts were analyzed. Of the 73 studies selected for reading in full, 21 were included: 15 preclinical and five clinical studies. The study selection process is summarized in the PRISMA flow diagram (Figure 1).

Figure 1. PRISMA flow diagram of the study selection process.

Preclinical studies

Sixteen included studies using animal models are presented in Table 1. Four studies were carried out in Brazil [17–20] and the United Kingdom [20–23]. Other studies were conducted in the USA [24,25], Iran [24,26,27] and Israel [28–30].

The *Leishmania* species evaluated in studies were *L (L.) major* (12), *L (L.) mexicana* (3) and *L. (L.) amazonensis* (2). Nguyen et al., 2019 [25]evaluated both *L. (L.) major* and *L. (L.) mexicana.* As expected, the Balb/c mouse (*Mus musculus*) was the most reported experimental model, and the infection was performed at the base of the animal's tail (13 studies).

Five studies evaluated the commercial drugs Fungizome, AmBisome®, Amphocil and SinaAmphoLeish 0.4% using the original formulation [21,24,29] or a modified formulation by the addition of adjuncts such as methylbenzethonium chloride, polylactic-co-glycolic acid, glycerol or ethanol [18,28]. Other studies evaluated new in-house formulations. Concerning the treatment route, eleven studies assessed the efficacy of a topically administered treatment (using a cream or drop formulation), followed by intralesional (2), subcutaneous (2) and intradermal administration (1). In general, the studies showed homogeneity in terms of the experimental model and species of *Leishmania* evaluated, site of infection and parameters for evaluation of treatment efficacy, which were systematically reported as the measure of the lesion size and parasitic load. However, an important difference in methodological approach was the time between the infection and the beginning of the treatment of the animals, varying from 24 hours [29] to 73 days after the infection [30]. This observation suggested that in some studies, the treatment was started even before the appearance of the lesion, which probably represents an important bias in this assessment.

The cure definitions adopted by the authors also varied significantly. For most studies, efficacy was presented not as the cure rate (the proportion of cured animals) but as the magnitude of the lesion size reduction and/or parasitological clearance (in the lesion

tissue and/or spleen). In general, wide variation was observed among the treatment protocols and outcomes reported, making it difficult to compare the results in these different studies.

Table 1. Main characteristics of the animal studies

Year, Author	Infection models			Treatment			Outcome		
	**Animal**	**Infection site/*Leishmania* species of infection**	**Animals/ group**	**Start of treatment**	**Intervention/administration route treatment schedule**	**Comparator**	**Methodology**	**Follow-up**	**Intervention X Comparator**
El-On, 1984	Balb/C	Tail base/ *L. (L.) major*	5	35 days post-infection	Fungisome with methyl benzethonium chloride/topical twice daily for 12 days	The lesion size before the treatment	~~Measurement of the~~ lesion size	30 days after end of treatment	There was no reduction in the lesion size or cure of any animals.
Yardley, 1997	Balb/C	Tail base/ *L. (L.) major*	NR	21 to 28 days post-infection	AmBisome®/subcutaneous 25 mg/kg once a day on six alternate days	Methylcellulose (negative control)	~~Measurement of the~~ lesion size	35 days after end of treatment	Nonsignificant reduction
Frankenburg, 1998	Mouse CBA	Tail base *L. (L.) major*	8	24 h post-infection	10 µl of Amphocil (2 mg/ml) topical, daily for 3 weeks; (I) in glycerol (II) in polypropylene glycol (III) in 10% ethanol (IV) in glucose	Untreated	~~Measurement of the~~ lesion size	35 days post-infection	Statistically significant reduction in the lesion size only for Amphocil with 10% ethanol
				24 h post-infection	10 µl of Amphocil (2 mg/ml) topical, daily for 3 weeks; (I) in 5% ethanol; (II) in 10% ethanol; (III) in 25% ethanol		measurement	28 days post-infection	Reduction in lesion size regardless of the ethanol
				24 h post-infection	(I) 10 µl of Amphocil (2 mg/ml) in 5% ethanol (topical), daily for 3 weeks; (II)10 µl of Fungizone (2 mg/ml) in 5% ethanol (topical), daily for 3 weeks; (III) 10 µl of Amphocil (2 mg/ml) in double-distilled water (topical), daily for 3 weeks;		measurement	28 days post-infection	The lesion size reduction with Fungizone is less than that with Amphocil.
Corware, 2010	Balb/C	Footpad/ *L. (L.) major*	24	7 days post-infection	AmB-polymethacrylic acid)/subcutaneous 2 mg/kg each day (7, 14 and 21 post infection day)	Water subcutaneous	~~Measurement of the~~ lesion size/lesion's parasitic count with a hemocytometer	80 days post-infection	35 days post-infection: 82 ± 2% lesion reduction and 99.85 ± 0.03% reduction in parasite viability/50 days post-infection: complete lesion healing/80 days post-infection: no relapse

Corware, 2011	Balb/C mice	Footpad/ *L. (L.) major*	18	7 days post-infection	AmB-polymethacrylic acid/intradermal 6 mg/kg each day (7, 14 and 21 post infection day)	Water	~~Measurement of the~~ lesion size/lesion's parasitic count with a hemocytometer	35 days post-infection	Resolution of cutaneous lesions and decrease in the parasite number by >3 log10 and 35 to 80 days post-infection: no relapse
			16	21 days post-infection	AmB-polymethacrylic acid/intradermal 6 mg/kg each day (21, 25 and 28 post infection day)			80 days post-infection	
Pinheiro, 2016	Balb/C	Tail base/ *L. (L.) major*	8	at least nodules in most animals and before ulcerative lesions	AmB 3% + Emulgel/topical 50 µL twice daily for 12 days	Emulgel topical	Measurement of the lesion size/lesion's parasitic load: no description of the methodology	35 days after the end of the treatment	Ulcerative lesions regressed gradually to even a complete cure/larger reduction in the number of recovered parasites.
					AmB 3% + Emulgel + Oleic Acid 5%/topical 50 µL twice daily for 12 days				Ulcerative lesions regressed gradually to even a complete cure/larger reduction in the number of recovered parasites.
Varikuti, 2017	Balb/C	Tail base/ *L. (L.) mexicana*	NR	56 days post infection	SinaAmphoLeish 0.4% (Nanoliposomal AmB)/topical twice daily for 28 days	Vaseline topical	~~Measurement of the~~ lesion size/lesion's parasitic load determined by limiting dilution	15-week post-infection	No significant differences in the lesion sizes and parasitic burdens
	Mouse 129SVE	Tail base/*L. (L.) mexicana*	NR	35 days post infection	SinaAmphoLeish 0.4% (Nanoliposomal AmB)/topical twice daily for 70 days	Vaseline topical	~~Measurement of the~~ lesion size/lesion's parasitic load determined by limiting dilution	15-week post-infection	Transient decrease in lesion sizes during the treatment but no significant differences in the lesion sizes and parasitic burdens
Abu Ammar, 2019	Balb/C	Tail base/ *L. (L.) major*	6 or 7	73 days post-infection	Poly(lactic-co-glycolic acid) nanoparticles (NPs) loaded with AmB deoxycholate/intralesional 1 mg/kg single dose	Control PBS; Blank NPs; AMB deoxycholate 1 mg/kg intralesional	~~Measurement of the~~ lesion size	34 days after star treatment	AMB NPs elicited a significantly greater lesion-reducing effect than the controls.
Jaafari, 2019	Balb/C	Tail base/ *L. (L.) major*	10	28 days post-infection	Nanoliposomal Amphotericin B 0.1% (50 mg) topical/twice daily for 28 days	PBS; Empty liposomes topical/twice daily for	~~Measurement of the~~ lesion size/lesion and spleen parasitic load	12-week post-infection	Remarkable reduction in the lesion size in treated animals from week 8 onward, with no significant difference among different groups

					Nanoliposomal AmB 0.2% (50 mg) topical/twice daily for 28 days	28 days	determined by limiting dilution		receiving AmB nanoliposomal/The splenic and lesion parasitic load in group treated with Lip-AmB 0.4% was significantly lower than control groups at week 12 post infection.
					Nanoliposomal AmB 0.4% (50 mg) topical/twice daily for 28 days				
Nguyen, 2019	Balb/C	Tail base/ *L. (L.) mexicana*	NR	35 days post-infection	AmB DMSO/topical 25 mg/kg/day once a day for 10 days	(I) PBS vehicle (II) AmBisome intraperitoneal 20 mg/kg	~~Measurement of the~~ lesion size/lesion´s parasitic load determined by limiting dilution	21 days after treatment	In lesion size significant differences in relation AmB dissolved in DMSO topical (p<0.05)/In the 500 µm needle group was the lowest parasitic load recorded in this experiment.
					AmB DMSO/topical with microneedling 500 µm 25 mg/kg/day once a day for 10 days				
				35 days post infection	AmB DMSO/topical microneedling 750 µm 25 mg/kg/day once a day for 10 days				
					AmB DMSO/topical with microneedling 1000 µm 25 mg/kg/day once a day for 10 days				
		Tail base/ *L. (L.) major*			AmB DMSO/topical 25 mg/kg/day once a day for 10 days	(I) Saline vehicle 0,9% intraperitoneal; (II) microneedles 750 µm individually; (III) AmBisome® intraperitoneal; topical AmB DMSO		14 days after treatment	There was no difference in the lesion size or parasitic load between the group receiving AmpB after microneedle and any of the non-AmBisome groups
					AmB DMSO/topical with microneedling 750 µm 25 mg/kg/day once a day for 10 days				
Sousa-Batista, 2019	Balb/C	Ear pinnae/ *L. (L.) amazonesis*	NR	10 or 30 days post-infection	Fungizone and polylactic-co-glycolic acid (PLGA)/intralesional/single dose 5 µg (0.2 mg/kg) of AmB in Day 10 (the model of early CL)	PBS	~~Measurement of the~~ lesion size; parasitic load; Measurement of the lesion size/lesion and lymph node parasitic load determined by limiting dilution	120 days after infection	At the end of fellow up, lesions were 37% smaller, and the parasite burdens in the ear and draining lymph nodes were 85% and 78% smaller than PBS control, respectively
			NR		Fungizone with polylactic-co-glycolic	PBS	~~measurement~~	90 days after	At the end of fellow up, lesions were 69%

					acid (PLGA)/intralesional/single dose 5 µg (0.2 mg/kg) of AmB in Day 30 (the model of established CL)		/lesion´s determined by limiting dilution	infection	smaller, and the parasite burdens in the ear and draining lymph nodes were 97% and 87% smaller than control, respectively
Alves, 2020	Balb/C	Tail base/ *L. (L.) major*	8	40 days post-infection	AmB 1,5% with gallic acid (GA) 1.5%/topical 50 µl twice daily for 21 days	AmB 3%; GA 3% and EA 3% each one topical with 50 µl twice daily for 21 days	~~Measurement of the~~ lesion size/lesion´s parasitic load determined by limiting dilution	14 days after the end of treatment	Amph B + GA and Amph B + EA had similar results to obtained with Amph B, GA, and EA/ Significant reduction in the parasitic load in animals treated with Amph B + GA e Amph B + EA
			8		AmB 1,5% with ellagic acid (EA) 1.5%/topical 50 µl twice daily for 21 days				
Dar, 2020	Balb/C	Tail base/ *L. (L.) mexicana*	5	28 days of infection	AmB-UDLs gel/topical 8 mg/kg, twice daily for 28 days	Untreated group (Carbopol gel topical) and simple AmB gel	~~Measurement of the~~ lesion size/lesion´s parasitic load determined by limiting dilution	28 days after the treatment	Significant reduction of the lesion size, but not completely resolved/ substantial reduction in the parasite burden
Fernández-Garciá, 2020	Balb/C	Tail base/ *L. (L.) amazonesis*	4	35 days of infection	AmB transfersomes vesicles (TFs)/topical 20 mg once daily for 10 days	Untreated animals (control group); Glucantime® (intralesionally 25 µL, 50 mg/kg)	~~Measurement of the~~ lesion size/lesion´s parasitic load determined by limiting dilution	56 days post-infection	The lesion size reduction was significant only 6 days after the end of the treatment/Decrease in the parasitic load is similar to that with intralesionally administered Glucantime.
Riaz, 2020	Balb/C	Tail base/ *L. (L.) major*	4	14 days of infection	AmB nanostructured lipid carriers (NLCs)/topical 50 µl for 10 days	Intravenous liposomal AmB (positive control) and no treatment (negative control)	~~Measurement of the~~ lesion size/lesion´s parasitic load determined by limiting dilution	24 days post-infection	No significant difference of the lesion size/significant reduction in the parasitic load compared to the negative control
Baharvandi, 2022	Balb/C	Tail base/ *L. (L.) major*	10	NR	AmB in microemulsion (ME) 0.4% topical, twice a day for 28 days (0.4 mg/day)	ME-Gel; AmB-Gel and Placebo topical/twice a day for 28 days	~~Measurement of the~~ lesion size/spleen´s parasitic load determined by Real - Time PCR	56 days post-infection	Remarkably smaller lesions/lower parasitic load compared to the placebo group.

NR: not reported; **PCR**: polymerase chain reaction; LDT: limiting dilution technique: MSL: measurement of the lesion size: **NPs PLGA**: poly(lactic-co-glycolic acid) nanoparticles; **PLGA**: poly(lactic-co-glycolic acid); **SGUDLs**: coloaded second generation ultradeformable liposomes; **NLCs**: nanostructured lipid carriers

Clinical studies

Only one randomized clinical trial ((RCT) was identified [31]. Among the other clinical studies, only one presented a control standard treatment group [32], while two studies compared two different AmB regimess [33,34] and one was not comparative [35]. The main methodological characteristics of the studies are presented in Table 2.

Four out five studies were performed in the Old World, three in Iran [31,32,35] and one in India [33]. Only one study was carried out in the Americas (Colombia) [34]. The number of treated patients ranged from 22 to 93 individuals. Intralesional infiltration [33,35] and topical [31,32,34] administration were evaluated in two and three studies, respectively. Two studies evaluated formulations produced by pharmaceutical companies, such as Humax Pharmaceutical S. A/Colombia [34] and Razaak Arak Pharmaceutical Company/Iran [32]. The other studies evaluated new formulations produced in-house.

All studies included patients with active skin lesions and CL confirmation based on direct examination, culture, or polymerase chain reaction (PCR). Only one study defined cure as complete re-epithelialization of all lesions and complete disappearance of the induration [34]. In other studies, the definition of cure was variable, in general assumed in face of 75 to 90% of epithelialization/ involution of the lesion [31,33,35]. In one study, the definition adopted for cure was not stated [32].

Only one study reported the relapse definition adopted: 100% re-epithelialization by Day 90 and subsequent emergence of lesions by Day 180 [34]. The time for cure

assessment varied widely among studies, ranging from 42 [32] to 240 days [31]. The follow-up length was in general also quite variable, ranging from 1.5 [32] to 13 months [35].

Table 2. Main characteristics of the human clinical studies

Author, year	Study design	Country (cases)	Treatment arms (patients)	Treatment scheme	CL case definition	Cure definition	Relapse definition	Cure assessment (days)	Follow-up (month)
Layegh, 2011	Comparative, randomized	Iran (n= 110)	Topical L-AmB [formulated from AmB deoxycholate] (50)	3–7 drops twice daily, for 8 weeks	Positive skin smear or biopsy of lesions within less than 6 months	75% decrease in the induration size	NR	D56	6 m
			Intralesional meglumine antimoniate [Glucantime] (60)	Once a week, until a fully infiltrated lesion to a maximum dose of 2 mL					
Goyonlo, 2014	Noncomparative, nonrandomized	Iran (n=93)	Intralesional AmB 2 mg/ml solution (93)	0.1 to 0.3 mL once a week, for up 13 weeks	Positive skin smear or lesion biopsy and history of antimony resistance or side effects	More than 90% reduction in inflammation and indurations	NR	D84	1-13 m
López, 2018	Noncomparative, nonrandomized	Colombia (n=80)	Topical AmB 3% [Anfoleish; H*umax Pharmaceutical S.A and PECET]* (80)	Topical three times daily, for 4 weeks	Positive skin smear or culture or PCR	Complete re-epithelialization of all ulcers and complete disappearance of the induration	Lesion that achieved 100% re-epithelialization by Day 90 that subsequently reopened by Day 180.	D90 and D180	6 m
				Topical twice daily, for 4 weeks					

Goswami, 2019	Noncomparative, nonrandomized	India (n=50)	Intralesional AmB solution 2.5 mg/ml (25)	Once a week, for 8 weeks	Positive skin smear for *Leishmania donovani* bodies	More than 90% reduction in size, induration, and ulceration; skin smear negative	NR	D84	6 m
			Intralesional AmB solution 5 mg/ml (25)	Once a week, for 8 weeks					
Khamesipour, 2022	Comparative, nonrandomized	Iran (n=52)	Topical nanoliposomes with AmB 0.4% [*Razaak Arak Pharmaceutical Company]* (22)	Twice daily, for 28 days	Positive direct smear, culture and PCR	NR	NR	D42	1,5 m
			intralesional meglumine antimoniate plus cryotherapy (30)	One injection per week (total of 7), plus biweekly cryotherapy (3 or 4 sessions)					

NR: not reported; **L- AMB**: liposomal amphotericin B; **AmB**: amphotericin B. **RCT**: randomized clinical trial; **IL:** intralesional

As shown in Table 3, the studies included predominantly adult patients, with an average age varying from 20.54 to 51 years. Overall, there was a balance between male and female participants in all studies [31–33,35], except in López et al., 2018 [34], where only male soldiers of the Colombian Army were included.

Most of the lesions were located on the head and neck [31,35], followed by the upper limbs in two studies [33,34]. One study did not report the lesions' location [32]. The duration of symptoms before treatment varied from 4 to 12 months.

Two studies identified the species of *Leishmania* [32,34]. Among them, *L. (V.) panamensis* was the most reported species (66/80) (López, 2018), followed by *L. (L.) major* and *L. (L.) braziliensis,* identified in 52/52 [32] and 12/80 [34], respectively (Table 3).

Table 3. Characteristics of the population treated with amphotericin B in human clinical studies

Author, year	Treatment arm/scam	Age (M_d ± SD or Age variation) years	Sex (male: female)	Duration of symptoms (months before therapy ± SD)	CL lesion site: n/N	Leishmania species characterization: n/N
Layegh, 2011	Topical L-AmB/twice daily	20.54 ± 18.72	23:27	1.06 ± 0.31	Head and neck: 26/50 (52%);<br>Hand: 18/50 (36%);<br>Leg and trunk: 6/50 (12%)	NR
	Intralesional meglumine antimoniate/once a week	25.30 ± 15.70	21:39	0.96 ± 0.43	Head and neck: 22/60 (36.6%);<br>Hand: 32/60 (53.3%);<br>Leg and trunk: 6/60 (10%)	NR
Goyonlo, 2014	Intralesional AmB/once a week	20.81 ± 15.26	44:49	<6: 19/93 (20.4%);<br>6-12: 46/93 (49.5%);<br>>12: 28/93 (30.1%)	Head and neck: 68/93 (73.1%);<br>Upper limb: 37/93 (39.8);<br>Lower limb and trunk: 17/93 (18.3%)	NR
López, 2018	Topical Anfoleish/3 times a day	24 (21–29 median)	39:1	NR	Head and neck: 6/40;<br>Thorax: 5/40;<br>Upper limbs: 25/40;<br>Lower limbs: 8/40	*L. (V.) braziliensis*: 6/40<br>*L. (V.) panamensis*: 33/40
	Topical Anfoleish/2 times a day	24 (21–29 median)	39:1	NR	Head and neck: 6/40;<br>Thorax: 2/40;<br>Upper limbs: 30/40;<br>Lower limbs: 6/40	*L. (V.) braziliensis*: 6/40<br>*L. (V.) panamensis*: 33/40
Goswami, 2019	Intralesional AmB/2.5 mg/ml once a week	33.00 ± 19.17	10:15	<6: 15/25 (60%)<br>>6: 10/25 (40%)	Head and neck: 11/25;<br>Upper limbs: 20/25;	NR

					Lower limbs and trunk: 9/25.	
	Intralesional AmB IL/5.0 mg/ml once a week	28.79 ± 17.08	16:9	<6: 18/25 (72%)<br>>6: 7/25 (28%)	Head and neck :9/25;<br>Upper limbs: 10/25;<br>Lower limbs and trunk: 9/25.	NR
Khamesipour, 2022	Topical L-AmB/twice a day	28–51 years	9:13	NR	NR	*L. (L.) major* 22/22
	Intralesional meglumine antimoniate/once a week	14-60	NR	NR	NR	*L. (L.) major* 30/30

NR: not reported; **nL-AMB**: Nanoliposomes of amphotericin B

The cure and relapse rates are shown in Table 4. Only Lopéz et al., 2018 [34] reported cure at D180, which would be assumed to be the definitive cure rate according to Oliaro et al., 2013 [13]. However, no difference was observed among the cure rates pooled at D90 and D180 [34]. The study of Goswami et al., 2019 [33], was the only investigation reporting the cure rate at D56 and D84. Here, we considered the cure rate reported at D84, as this timepoint was the closest to D90.

For topical administration of AmB, the cure rates ranged from 30% [34] to 81.8% [32] (Table 4), and the pooled cure rate was 45.6% [CI: 27.5–64.8%; I^2 = 79.6) (Figure 2a). For studies addressing AmB intralesional infiltration, the cure rate ranged from 61.3% [35] to 88% [33] (Table 4), and the pooled cure rate was 69.8% [CI: 52.3–82.9%; I^2 = 63.9) (Figure 2b), notably superior to that for topical treatment with AmB. The overall cure rate at D90 for all local interventions gathered (topical and intralesional AmB) was 56.9% [CI: 41.1–71.4%; I^2 = 82.8], as shown in Figure 2c.

Two trials directly compared topical AmB treatment with meglumine antimoniate intralesional infiltration, allowing meta-analysis. Assessing 162 patients and considering cure at D90, no difference was observed between interventions (OR: 1.7; 0.34–9.15, I^2 = 79,1) (Figure 3).

Table 4. Cure and relapse rates according to the intent-to-treat approach (human clinical studies)

Author, year	Treatment arm/scam	Initial cure rate (D90) n/N (%)	Relapse rate % (at 6 months)	Adverse effects (n/N)
Layegh, 2011	Topical L-AmB/twice daily	D56 = 22/50 (44)	0/22	Mild pruritus around the lesions (5/50)
	Intralesional meglumine antimoniate/once a week	D56 = 29/60 (48.3)	0/22	Erythema and edema at the injection site (7/60); Hypersensitivity (1/60)
Goyonlo, 2014	Intralesional AmB/2 mg/mL once a week	D84=57/93 (61.4)	4/57	Local pain during injection (93/93); Prolonged pain (>30 min) (17/93); Fibrosis at the injection site (12/93); Local allergic reaction (1/93)
López, 2018	Topical Anfoleish/3 times a day	D90=12/40 (30)	0/40	Burning sensation, itching and rash (5/40); Mild and transitory elevation of transaminases (2/40) or creatinine (1/40); varicella (1/40); flu (7/40); muscle aches (6/40); gastrointestinal symptoms (10/40).
	Topical Anfoleish/twice daily	D90=13/40 (32.5)	0/40	Burning sensation, itching and rash (2/40)
Goswami, 2019	Intralesional AmB/2.5 mg/ml once a week	D84=22/25 (88)	0/25	Pain during injection after no more than 30 min (25/25)
	Intralesional AmB IL/5.0 mg/ml once a week	D84=16/25 (64)	0/25	
Khamesipour, 2022	Topical L-AmB/twice a day	D42 = 18/22 (81.8)	NR	Burning sensation (2/22)

	Intralesional meglumine antimoniate	D42=15/30 (50)	NR	NR

NR: not reported.

Figure 2. Pooled cure rate including all patients treated with topical amphotericin B (a), intralesional amphotericin B (b) and all local treatments (c).

Figure 3. Meta-analysis of studies directly comparing topical amphotericin B treatments and intralesional meglumine antimoniate

Adverse events

Of the five clinical studies evaluated, four reported the occurrence of adverse events (AEs). Based on studies with available adverse event data, we identified 195 AEs among 290 treated patients. For topical treatment, a burning sensation, mild itching around the lesions and rash were the reported adverse effects. In the studies addressing AmB intralesional infiltration, all patients reported local pain during injections (Table 4).

Risk of bias in the included studies

The assessment of risk of bias is shown in Figure 4. Regarding preclinical studies, considerable homogeneity was observed. However, a high risk of bias was confirmed for selection, performance and detection domains, given the absence of random allocation of animals and lack of blinding during cure assessment. A low risk of bias was observed in relation to the description of the result (Figure 4a).

Regarding nonrandomized clinical studies, it has been observed that the absence of a comparator is the factor that most impacts bias, affecting the domains of selection and

comparability. Regarding the results, in general, a low risk of bias was verified (Figure 4b). The only one randomized clinical trial [31] was assessed using Rob 2.0 tool, with a high risk of bias being observed for most domains evaluated and some concerns regarding the randomization process and measurement of results (Figure 3).

Figure 4. Risk of bias observed in the included studies.

DISCUSSION

The first finding of this review was confirmation of the still limited experience with locally administered AmB interventions for CL: there are only 16 preclinical and five human studies published between 1984 and 2022. In addition to scarcity, the *Leishmania* species prevalent in Old World countries are more represented in the retrieved studies, and, in general, there is a lack of alignment between preclinical and clinical studies, that evaluated very different interventions, reflecting the absence of a development plan for local treatment with AmB.

Treatment is still considered the main strategy for leishmaniasis control, given the lack of vaccines and difficulties in implementing actions focused on the vector. However, the therapeutic arsenal available is limited, either because of lack of investment in tropical infectious diseases or because of challenges related to the discovery and development of new effective drugs against this intracellular parasite. The local or systemic treatments currently recommended by the World Health Organization (WHO) and Pan American Health Organization (PAHO) are marked by many inconveniences, such as parenteral use, long-term treatments and high toxicity, whether hepatic, cardiac,

metabolic or renal [36]. In this scenario, the repositioning and evaluation of alternative routes of administration of existing drugs emerges as a potentially useful strategy to expand the therapeutic options for leishmaniasis, aiming to overcome such limitations.

The switch in the antimony administration route in recent years represents a successful case of drug safety profile improvement in the CL field. With a cure rate similar to that observed with parenteral use [9], the intralesional infiltration approach is linked to a greater schedule flexibility and a low risk of high-intensity or severe complications. Based on this rationale, this review was proposed as the preparatory step for a future complete development plan for the local use of AmB, gathering existing preclinical and clinical trials.

Layegh et al. (2011) [31] demonstrated how a set of evidence obtained in preclinical trials [29] can pave the way for clinical use. In the case of a new topical nanoliposomal AmB formulation, promising results of stability, diffusion and efficacy were obtained in one preclinical investigation [26] before phase 1 and 2 clinical studies [32], a progressive and orderly construction of evidence not always seen.

The consolidation of the CL therapeutic modality based on antimony infiltration [36] possibly contributed to the increased interest in the local use of AmB. In this sense, preclinical trials represent an ideal starting point for the exploration of different formulations and schemes [23,30] in different populations [18].

These results confirm that local use of AmB, in its different formulations, has already been studied in animal and clinical trials. The most analyzed AmB formulations are commercially available compounds (deoxycholate and liposomal) associated with different vehicles, such as gel and ointment. Although there are 89 CL-endemic countries distributed throughout the Eastern Mediterranean region, the Americas and Africa [37], most of the studies carried out have been performed in the Old World, where species such as *L. (L.) major* and *L. (L.) tropica* are predominant.

In addition to this low world species representativity, the lack of methodological standardization and the qualitative approach adopted by most preclinical studies prevent us from pooling the results in meta-analysis, even to compare them. It is important to highlight the existence of a recently developed tool to standardize animal studies and ensure that they are reported in sufficient detail [38]. One of the most important differences among studies is the time of initiation of treatment, apparently before lesion onset, in some studies.

Regarding clinical trials, most are not comparative, involving a small number of patients and presenting a high risk of bias in different domains. However, some useful observations can be drawn from the experiences gathered thus far. It can be highlighted, among others, the observation of the lack of association between effect and number of applications of the ointment formulation addressed by López at al., (2018) [34], in the same way that a higher concentration of the amphotericin solution infiltrated by Goswami et al., (2019) [33] did not affect the efficacy of the treatment.

Two topical interventions yielded therapeutic success in less than half of treated patients [31,34], while the intervention addressed by Khamesipour et al., (2022) [32] reached a cure rate of 81.8%. This discrepancy in results suggests that there may be a difference between formulations or, alternatively, it may have also been influenced by the cure definition adopted in the studies, not always clearly presented in the publication.

It is important to point out that, in this comparison of extreme cure rates, the study with the highest cure rate addressed a total of 22 participants infected with *L. (L.) major* while the one with the lowest efficacy was conducted by López et al., (2018) [34], in Colombia, comprising 80 patients infected with *L. (L.) panamensis* and *L. (V.) braziliensis.* In addition, this Colombian study used the most demanding definition of cure, namely, "complete re-epithelialization of the ulcers". Another noteworthy aspect is the differences between the spontaneous cure rates in the Old and New World, estimated at 60% for species such as *L. (L.) major* [39] and approximately 6% for *L. (V.) braziliensis* [40]. Although we cannot assume that the differences are due to *Leishmania* species, considering the lack of reporting of this information, it is possible to infer that different species may have contributed to the heterogeneity observed in these results.

Even if only indirectly, a comparison of the results of the studies needs to be evaluated with caution, given the enormous methodological differences between them. For example, Goswami et al., 2019 [33] and Goyolo et al., 2014 [35], despite having used similar schemes in terms of interval and dose (AmB 2.5 mg/ml once a week), presented different results, which may be related to the different *Leishmania* species present in Iran and India, respectively.

Evidence thus far accumulated on local AmB treatment demonstrates the viability of this route of administration. Based on the studies gathered here, it is not possible to identify a formulation or a specific route for local amphotericin administration that is superior to the others. Among all the evaluated alternatives, the intralesional infiltration strategy based on the already commercialized amphotericin would be the faster option, considering the entire chain of development and production already completed. In addition to efficacy data, future studies should include pharmacokinetics and safety parameters to produce a complete and consistent set of information to support the proposition of new therapeutic regimens for leishmaniasis.

ACKNOWLEDGEMENTS

We are grateful for support from the Programa de Pós Graduação em Ciências da Saúde of the Instituto René Rachou; Coordination for the Improvement of Higher Education Personnel (CAPES); National Council for Scientific and Technological Development (CNPq: 302069/2022-4).

FUNDING

This study was partially supported by the Coordenação de Aperfeiçoamento de Pessoal de Nível Superior - CAPES - Finance Code 001. Conselho Nacional de Desenvolvimento Científico e Tecnológico, grant number 302069/2022-4 to Gláucia Cota.

REFERENCES

1. PAHO. Leishmanioses: Informe Epidemiológico das Américas, No. 10 (Dezembro 2021). OPAS; 2021 Dec. Available: https://iris.paho.org/handle/10665.2/55386

2. WHO. Global leishmaniasis surveillance: 2021, assessing the impact of the COVID-19 pandemic. 2022 [cited 5 Jun 2023]. Available: https://www.who.int/publications/i/item/who-wer9745-575-590

3. de Castro Toledo AC, da Silva RE, Carmo RF, Amaral TA, Profeta Luz Z lia M, Rabello A. Assessment of the quality of life of patients with cutaneous leishmaniasis in Belo Horizonte, Brazil, 2009-2010. A pilot study. Trans R Soc Trop Med Hyg. 2013;107: 335–336. doi:10.1093/TRSTMH/TRT021

4. Bennis I, Belaid L, De Brouwere V, Filali H, Sahibi H, Boelaert M. “The mosquitoes that destroy your face”. Social impact of Cutaneous Leishmaniasis in South-eastern Morocco, A qualitative study. PLoS One. 2017;12: e0189906. doi:10.1371/JOURNAL.PONE.0189906

5. Castro M del M, Rode J, Machado PRL, Llanos-Cuentas A, Hueb M, Cota G, et al. Cutaneous leishmaniasis treatment and therapeutic outcomes in special populations: A collaborative retrospective study. PLoS Negl Trop Dis. 2023;17: e0011029.

6. Laniado-Laborín R, Cabrales-Vargas MN. Amphotericin B: side effects and toxicity. Rev Iberoam Micol. 2009;26: 223–227.

7. Vyas SP, Gupta S. Optimizing efficacy of amphotericin B through nanomodification. Int J Nanomedicine. 2006;1: 417–432.

8. Hamill RJ. Amphotericin B formulations: a comparative review of efficacy and toxicity. Drugs. 2013;73: 919–934.

9. Brito NC, Rabello A, Cota GF. Efficacy of pentavalent antimoniate intralesional infiltration therapy for cutaneous leishmaniasis: A systematic review. PLoS One. 2017;12: e0184777. doi:10.1371/JOURNAL.PONE.0184777

10. Carneiro G, Aguiar MG, Fernandes AP, Ferreira LAM. Drug delivery systems for the topical treatment of cutaneous leishmaniasis. Expert Opin Drug Deliv. 2012;9: 1083–1097.

11. Liberati A, Altman DG, Tetzlaff J, Mulrow C, Gøtzsche PC, Ioannidis JPA, et al. The PRISMA statement for reporting systematic reviews and meta-analyses of studies that evaluate health care interventions: explanation and elaboration. Ann Intern Med. 2009;151: W-65.

12. Ouzzani M, Hammady H, Fedorowicz Z, Elmagarmid A. Rayyan-a web and mobile app for systematic reviews. Syst Rev. 2016;5: 1–10. doi:10.1186/S13643-016-0384-4/FIGURES/6

13. Olliaro P, Vaillant M, Arana B, Grogl M, Modabber F, Magill A, et al. Methodology of clinical trials aimed at assessing interventions for cutaneous leishmaniasis. PLoS Negl Trop Dis. 2013;7: e2130.

14. Hooijmans CR, Rovers MM, De Vries RBM, Leenaars M, Ritskes-Hoitinga M, Langendam MW. SYRCLE's risk of bias tool for animal studies. BMC Med Res Methodol. 2014;14: 1–9.

15. Ottawa Hospital Research Institute. [cited 5 Jun 2023]. Available: https://www.ohri.ca/programs/clinical_epidemiology/oxford.asp

16. Higgins JPT, Thomas J, Chandler J, Cumpston M, Li T, Page MJ, et al. Cochrane handbook for systematic reviews of interventions. John Wiley & Sons; 2019.

17. Pinheiro IM, Carvalho IP, de Carvalho CES, Brito LM, da Silva ABS, Júnior AMC, et al. Evaluation of the in vivo leishmanicidal activity of amphotericin B emulgel: An alternative for the treatment of skin leishmaniasis. Exp Parasitol. 2016;164: 49–55.

18. Sousa-Batista AJ, Pacienza-Lima W, Ré MI, Rossi-Bergmann B. Novel and safe single-dose treatment of cutaneous leishmaniasis with implantable amphotericin B-loaded microparticles. Int J Parasitol Drugs Drug Resist. 2019;11: 148–155.

19. Alves MM de M, Arcanjo DDR, Figueiredo KA, Oliveira JS de SM, Viana FJC, Coelho E de S, et al. Gallic and ellagic acids are promising adjuvants to conventional amphotericin B for the treatment of cutaneous leishmaniasis. Antimicrob Agents Chemother. 2020;64: e00807-20.

20. Fernández-García R, Statts L, De Jesus JA, Dea-Ayuela MA, Bautista L, Simão R, et al. Ultradeformable lipid vesicles localize amphotericin B in the dermis for the treatment of infectious skin diseases. ACS Infect Dis. 2020;6: 2647–2660.

21. Yardley V, Croft SL. Activity of liposomal amphotericin B against experimental cutaneous leishmaniasis. Antimicrob Agents Chemother. 1997;41: 752–756.

22. Corware KD, Rogers M, Teo I, Müller I, Shaunak S. An amphotericin B-based drug for treating experimental Leishmania major infection. Trans R Soc Trop Med Hyg. 2010;104: 749–750.

23. Corware K, Harris D, Teo I, Rogers M, Naresh K, Müller I, et al. Accelerated healing of cutaneous leishmaniasis in non-healing BALB/c mice using water soluble amphotericin B-polymethacrylic acid. Biomaterials. 2011;32: 8029–8039.

24. Varikuti S, Oghumu S, Saljoughian N, Pioso MS, Sedmak BE, Khamesipour A, et al. Topical treatment with nanoliposomal Amphotericin B reduces early lesion growth but

fails to induce cure in an experimental model of cutaneous leishmaniasis caused by Leishmania mexicana. Acta Trop. 2017;173: 102–108.

25. Nguyen AK, Yang K-H, Bryant K, Li J, Joice AC, Werbovetz KA, et al. Microneedle-based delivery of Amphotericin B for treatment of cutaneous Leishmaniasis. Biomed Microdevices. 2019;21: 1–10.

26. Jaafari MR, Hatamipour M, Alavizadeh SH, Abbasi A, Saberi Z, Rafati S, et al. Development of a topical liposomal formulation of Amphotericin B for the treatment of cutaneous leishmaniasis. Int J Parasitol Drugs Drug Resist. 2019;11: 156–165.

27. Baharvandi Z, Salimi A, Arjmand R, Jelowdar A, Rafiei A. Evaluation of the Efficacy of Amphotericin B and Terbinafine Microemulsions and Their Combination on Cutaneous Leishmaniasis and Comparison with the Conventional Drug Form in BALB/c Mice. AAPS PharmSciTech. 2022;23: 280.

28. El-On J, Jacobs GP, Witztum E, Greenblatt CL. Development of topical treatment for cutaneous leishmaniasis caused by Leishmania major in experimental animals. Antimicrob Agents Chemother. 1984;26: 745–751.

29. Frankenburg S, Glick D, Klaus S, Barenholz Y. Efficacious topical treatment for murine cutaneous leishmaniasis with ethanolic formulations of amphotericin B. Antimicrob Agents Chemother. 1998;42: 3092–3096.

30. Abu Ammar A, Nasereddin A, Ereqat S, Dan-Goor M, Jaffe CL, Zussman E, et al. Amphotericin B-loaded nanoparticles for local treatment of cutaneous leishmaniasis. Drug Deliv Transl Res. 2019;9: 76–84.

31. Layegh P, Rajabi O, Jafari MR, Emamgholi Tabar Malekshah P, Moghiman T, Ashraf H, et al. Efficacy of topical liposomal amphotericin B versus intralesional meglumine antimoniate (Glucantime) in the treatment of cutaneous leishmaniasis. J Parasitol Res. 2011;2011.

32. Khamesipour A, Mohammadi A, Jaafari M, Eskandari S, Tasbihi M, Javadi A, et al. Pilot study of safety and efficacy of topical liposomal amphotericin B for cutaneous leishmaniasis caused by Leishmania major in Islamic Republic of Iran. East Mediterr Health J. 2022;28: 658–663.

33. Goswami P, Ghiya BC, Kumar V, Rekha S, Mehta RD. Comparison of efficacy of two different concentrations of intralesional amphotericin B in the treatment of cutaneous leishmaniasis; A randomized controlled trial. Indian Dermatol Online J. 2019;10: 627.

34. López L, Vélez I, Asela C, Cruz C, Alves F, Robledo S, et al. A phase II study to evaluate the safety and efficacy of topical 3% amphotericin B cream (Anfoleish) for the treatment of uncomplicated cutaneous leishmaniasis in Colombia. PLoS Negl Trop Dis. 2018;12: e0006653.

35. Goyonlo VM, Vosoughi E, Kiafar B, Nahidi Y, Momenzadeh A, Taheri AR. Efficacy of intralesional amphotericin B for the treatment of cutaneous leishmaniasis. Indian J Dermatol. 2014;59: 631.

36. PAHO. Guideline for the Treatment of Leishmaniasis in the Americas. Second Edition. Guideline for the Treatment of Leishmaniasis in the Americas Second Edition. Pan American Health Organization; 2022. doi:10.37774/9789275125038

37. WHO. 2023 [cited 5 Jun 2023]. Available: https://www.who.int/health-topics/leishmaniasis#tab=tab_1

38. du Sert NP, Hurst V, Ahluwalia A, Alam S, Avey MT, Baker M, et al. The ARRIVE guidelines 2.0: Updated guidelines for reporting animal research. PLoS Biol. 2020;18: e3000410. doi:10.1371/JOURNAL.PBIO.3000410

39. Bailey MS, Lockwood DNJ. Cutaneous leishmaniasis. Clin Dermatol. 2007;25: 203–211.

40. Cota GF, de Sousa MR, Fereguetti TO, Saleme PS, Alvarisa TK, Rabello A. The cure rate after placebo or no therapy in American cutaneous leishmaniasis: a systematic review and meta-analysis. PLoS One. 2016;11: e0149697.

SUPPORTING INFORMATION CAPTIONS

S1 Table. PRIMA Checklist

S2 Table. Search strategies

Fig 1

Click here to access/download;Figure;Fig 1.tif

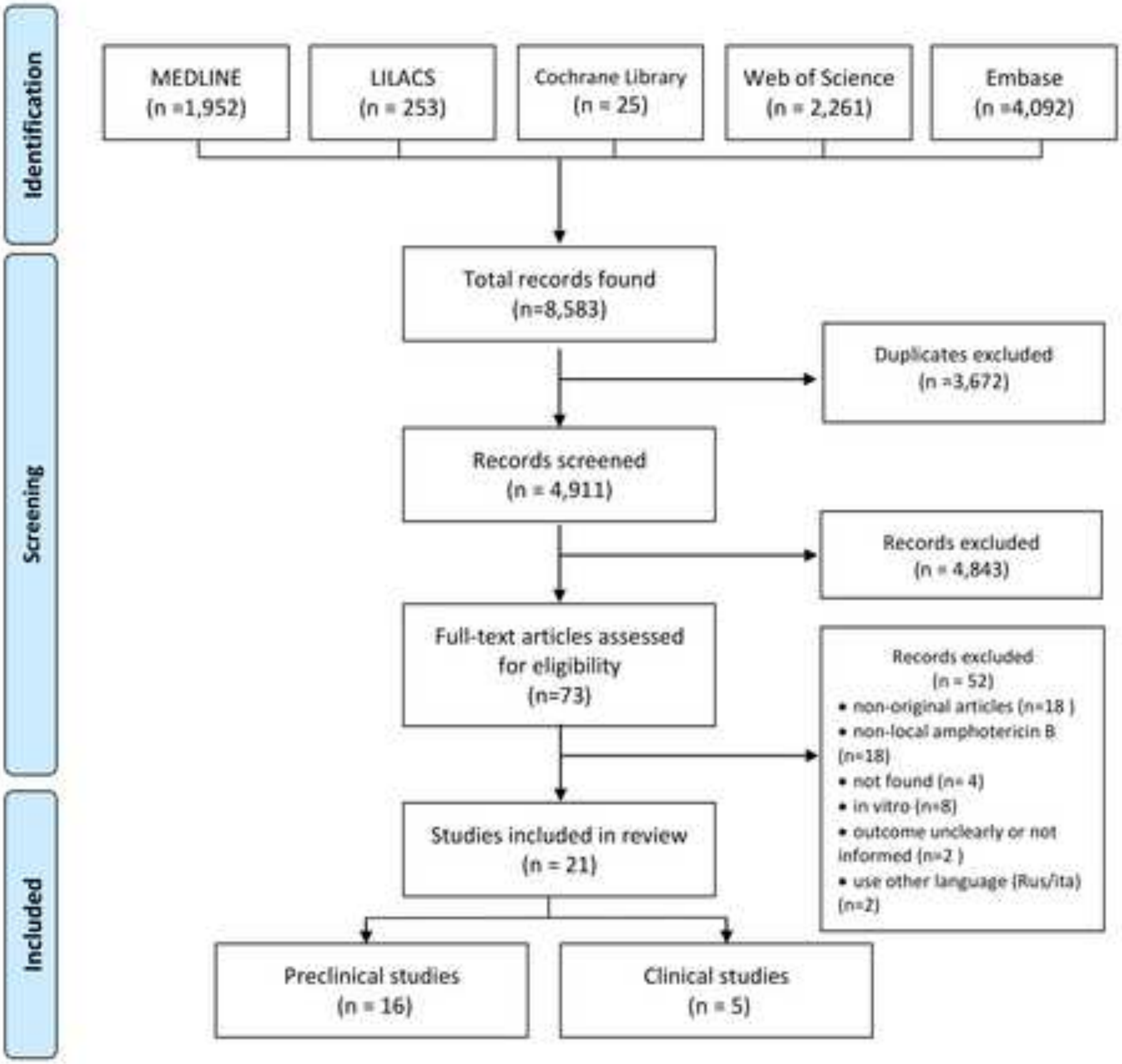

Fig 2

Click here to access/download;Figure;Fig 2.tif

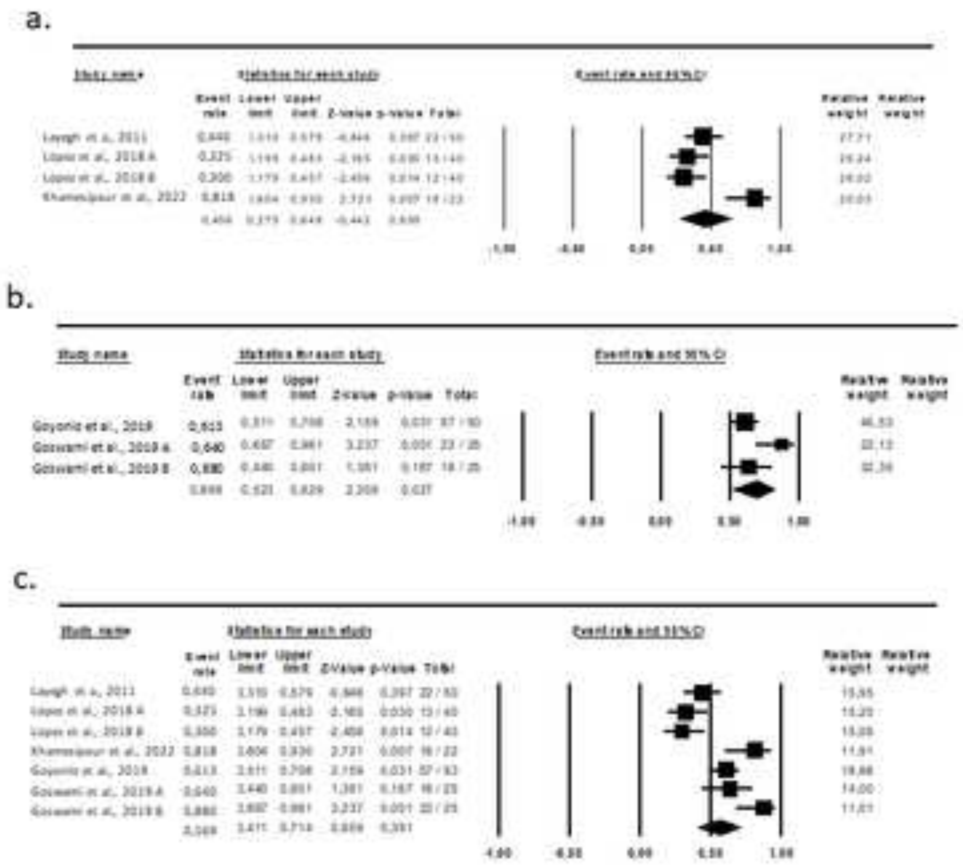

Click here to access/download;Figure;Fig 3.tif

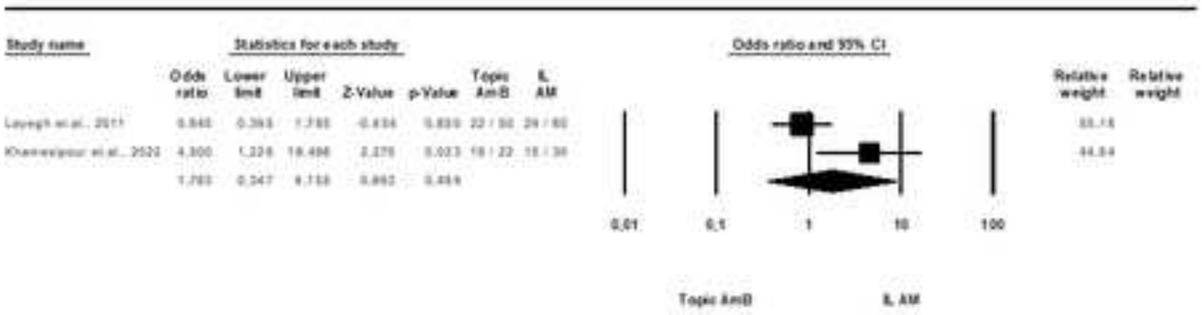

Fig 4

Click here to access/download;Figure;Fig 4.tif

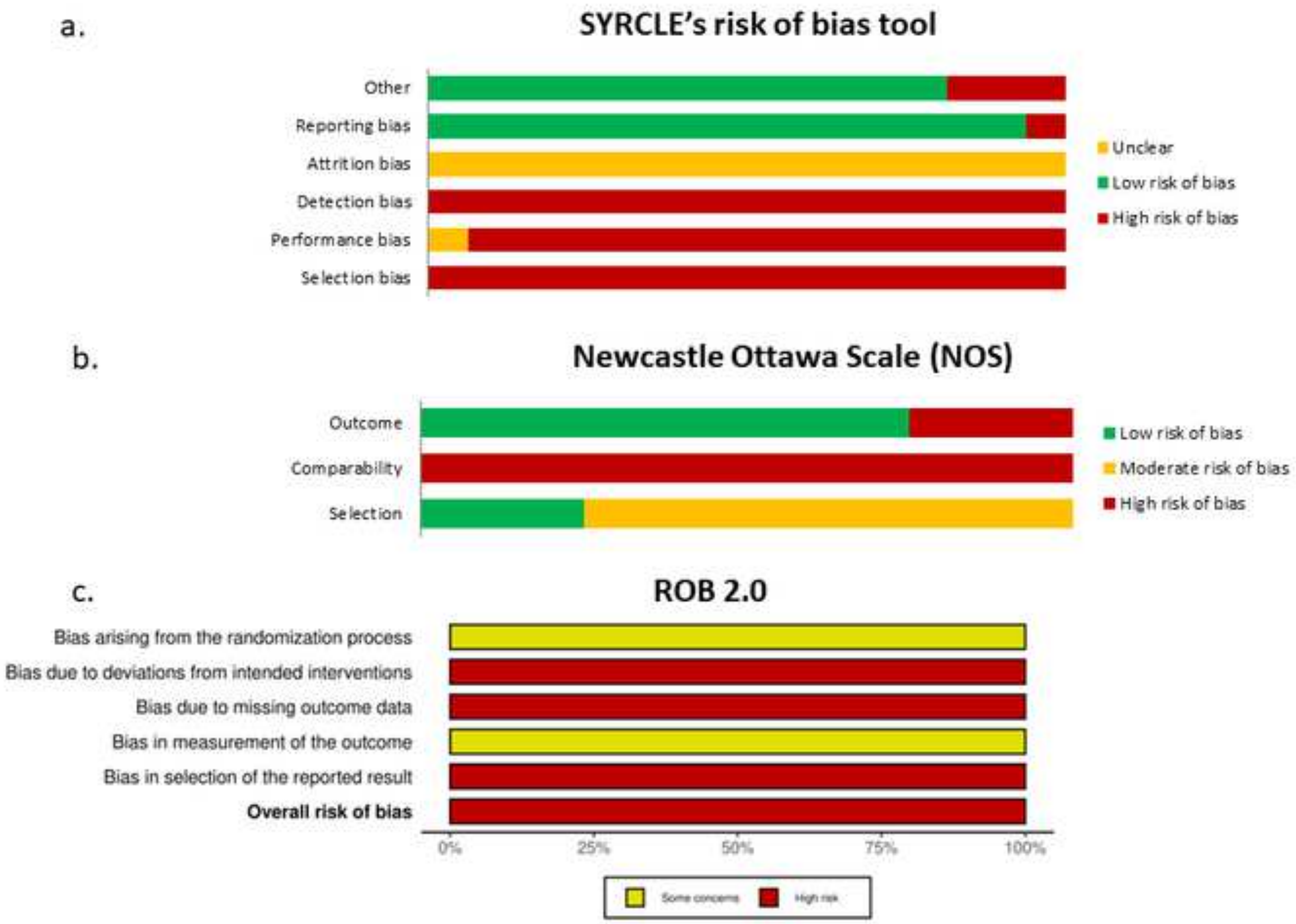

Click here to access/download
Supporting Information
S1 Table.docx

Click here to access/download
Supporting Information
S2 Table.docx

English Certificate

Editing Certificate

This document certifies that the manuscript

LOCAL AMPHOTERICIN B THERAPY FOR CUTANEOUS LEISHMANIASIS: A SYSTEMATIC REVIEW

prepared by the authors

Líndicy Leidicy Alves; Mariana Lourenço Freire; Isadora Lana Troian; Eliane de Morais-Teixeira; Gláucia Cota

was edited for proper English language, grammar, punctuation, spelling, and overall style by one or more of the highly qualified native English speaking editors at AJE.

This certificate was issued on **June 2, 2023** and may be verified on the AJE website using the verification code **7A4A-1A5B-9D7C-AD94-4BEE**.

Neither the research content nor the authors' intentions were altered in any way during the editing process. Documents receiving this certification should be English-ready for publication; however, the author has the ability to accept or reject our suggestions and changes. To verify the final AJE edited version, please visit our verification page at aje.com/certificate. If you have any questions or concerns about this edited document, please contact AJE at support@aje.com.

AJE provides a range of editing, translation, and manuscript services for researchers and publishers around the world. For more information about our company, services, and partner discounts, please visit aje.com.
