# Supplementary material for: Local amphotericin B therapy for Cutaneous Leishmaniasis: A systematic review
Source: PLoS Negl Trop Dis. 2024 Apr 16;18(4):e0012127. doi: 10.1371/journal.pntd.0012127 (PMC11051593; doi:10.1371/journal.pntd.0012127)
Supplement: Attachment — Submitted filename: Response to Review.docx [file pntd.0012127.s006.docx]

December 01, 2023

Dear Dra. Ana Laura Grossi de Oliveira,

We are grateful to the reviewers for their time and valuable contributions to improve the manuscript **"Local amphotericin B therapy for cutaneous leishmaniasis: a systematic review"** (PNTD-D-23-00751). We have carefully considered all reviewers’ concerns and did our best to improve the text accordingly. In all cases we’ve included in the manuscript the required changes. The modifications implemented in this revised version are summarized below together with specific answers to the questions placed by the reviewers.

Please let us know in case any other improvement in the manuscript becomes necessary.

Sincerely,

Líndicy Leidicy Alves

lindicy.leidicy@hotmail.com

**Reviewer #1:**

1. **I have recommended the GRADE to evaluate the certainty of evidence.**

*Authors’ comment:* We thank the reviewer for giving us the opportunity to discuss this important point. The GRADE (Grading of Recommendations Assessment, Development, and Evaluation) tool aims to grade the quality of evidence supporting health recommendations. Only exploratory studies evaluating amphotericin B in local administration were found in this review. These results cannot support health recommendations but must be used to encourage new guides and better-designed studies. This is why we haven’t presented the GRADE assessment in our first manuscript version, what we have done now (Supplementary Table 4) together with the appropriate comments on the care to be taken when interpreting these results (lines 405-407).

2. **The figures are not of sufficient quality for clarity and need to be improved.**

*Authors’ comment:* Thank you for your comment We checked the Plos Neglected Tropical Diseases author guidelines, and all necessary modifications were made to meet publication requirements.

3. **The authors must consider the GRADE analysis for conclusion.**

*Authors’ comment:* Thank you for your suggestion, this point has been incorporated into the 405-407 lines.

**4. Line 37-38: Since the percentage cure is about 57% (reasonable), and considering the risk of bias, inconsistencies, and imprecision, the authors should be cautious in recommending this treatment. The GRADE is recommended.**

*Authors’ comment:* Again, it is a very important point to be discussed. According to the systematic review by Brito et al., 2017 (DOI: 10.1371/journal.pone.0184777), the cure rate with intralesional pentavalent antimony in the New World from randomized clinical trials was 63.2% (IC95 % 52.8–72.6%). This is the main treatment recommendation for patients with localized cutaneous leishmaniasis caused by *L. (L.) braziliensis* and *L. (V.) amazonensis* (OPAS, 2022 https://doi.org/10.37774/9789275725030). This observation supports the statement that the estimated cure rates (57%) gathered so far with locally administrated amphotericin B is sufficient to encourage the continuity of research with this therapeutic approach. This perspective was included in our rational in the manuscript.

**5. Line 42: Choose a Mesh term in Pubmed.**

*Authors’ comment: The* mesh terms were considered to define the keywords.

**6. Line 104: When was the protocol update?**

*Authors’ comment:* The protocol in PROSPERO was updated.

**7. Line 104-105: Give the PICO question.**

**8. Line 120: Was the PICO acronym used to apply the criteria?**

*Authors’ comment:* The PICO question and the inclusion criteria using PICO acronym were presented more clearly in the topic Eligibility criteria on the lines 99-114.

**9. Line 163-164: When was the inconsistency considered substantial/significant?**

*Authors’ comment:* The interpretation of heterogeneity was done according to the Cochrane Handbook, and it has now been presented in the lines 154-157.

**10. Line 166: The level of evidence assessment by GRADE is recommended for human and animal studies.**

*Authors’ comment:* Again, we do agree with the reviewer about the need to have the quality of evidence assessed by the Grade system, which has recently also been explored for animal studies for situations where pre-clinical results are contributing to support the formulation of evidence-based policies, in the absence of results in humans (WEI, Dang et al. 2016. DOI: https://doi.org/10.1111/jebm.12198). However, in this present case, there two reasons to not perform GRADE assessment for animal studies: I. the lack of quantitative results summarizing the direction of the effect of the interventions and ii. The lack of compliance with the requirement, at this stage, we are dealing with evidence supporting the recommendation for human use. Thus, according to our understanding, these aspects preclude a meaningful assessment of the certainty of the evidence from animal studies using the GRADE framework.

**11. Line 167: How or who resolved the discrepancies?**

*Authors’ comment:* The discrepancies in the studies' quality assessment were resolved through consensus, or by a third reviewer (GC) in case of the absence of consensus. This information was included in the lines 168-169

**12. Line 174: All figures require resolution improvements.**

*Authors’ comment:* Thank you for your suggestion. We checked the Plos Neglected Tropical Diseases author guidelines, and all necessary modifications were made to meet publication requirements.

**13. Line 180: Give a list of excluded references (n=52) as a supplementary file.**

*Authors’ comment:* The list of excluded references was created and can be found in supplementary table 3.

**14. Table 1: Table has no vertical lines.**

*Authors’ comment:* Thank you for your observation. The correction was made in all tables.

**15. Table 1: Give the reference number for all citations.**

*Authors’ comment:* The reference number of each citation was reported in all table.

**16. Table 1: How was it measured?**

*Authors’ comment:* The correction was made in the table.

**17. Line 258: To include p-value.**

*Authors’ comment:* P-value was included for each pooled cure rate in the text and in each figure.

**18. Line 267: It is necessary to improve the quality of all figures since this impaired the article revision.**

*Authors’ comment:* Thank you for your observation. We checked the Plos Neglected Tropical Diseases author guidelines, and all necessary modifications were made to meet publication requirements.

**19. Line 275: Give citations.**

**20. Line 277-278: Report the dose tested that showed this effect.**

*Authors' Comment:* The section dedicated to the adverse events has been revised, resulting in a clear presentation of this information (Lines 268-283).

**21. Line 298: Since nanoparticle has been used in some studies, the authors did not explore this promisor topic in discussion. Maybe these studies show better results than the other formulations.**

*Authors’ comment:* Thank you for this suggestion. This aspect was now included in the discussion (lines 352-359), as following: Amphotericin B associated with nanoparticles has been assessed in four preclinical trials (Varikuti, 2017; Abu Ammar, 2019; Jaafari, 2019; Riaz, 2020). Among these, only the study conducted by Abu Ammar in 2019, which evaluated poly(lactic-co-glycolic acid) nanoparticles loaded with amphotericin B deoxycholate, demonstrated a reduction in lesion size compared to controls. In other studies, nanoparticles associated with amphotericin B failed to induce a statistically significant reduction in lesion size compared to their respective controls (Varikuti, 2017; Jaafari, 2019; Riaz, 2020). In the clinical trial led by Khamesipour in 2022, the results were notably promising, revealing a cure rate of 81.8% (18/22) for topical treatment with nanoliposomes containing 0.4% amphotericin B. However, this study involved a limited number of patients (n=22) infected by *L. (L.) major*, a Leishmania species related to a relatively high rate of spontaneous cure.

**22. Line 373: This is a perfect definition of wound healing/cure, since that it is the dreamer result for patients. Only 75-90% of efficacy will not solve your problem. So considering that only one study obtained 100% of re-epithelization, we are so far that is good.**

*Authors’ comment:* The lack of harmonization of outcomes is one of the issues identified in the literature. It is important to highlight that for clinical trials in cutaneous leishmaniasis, there is a published consensus that recommends the use of 100% epithelialization as the criterion for evaluating cure (Olliaro et al, 2013). Among the studies retrieved in this review, only one study used this criterion. For this reason, we chose to present the outcome as reported by the authors of the primary studies, a limitation discussed in the manuscript.

**Reviewer #2:**

**1.	The introduction of the study and its goal is clear. The methods are also clearly defined and in line with reporting guidelines for systematic reviews. There are, however, some concerns about the decision to conduct a meta-analysis and estimate pooled cure rates with very heterogeneous studies. Authors also described having to "arbitrarily assumed as the initial cure rate that assessed between D42 and D90." Why did the authors decide for this approach instead of a qualitative synthesis of the available literature?**

*Authors’ comment:* Based on the current harmonized criteria for CL trials (Olliaro, 2013), the choice of the interval D45 to D90 as the time for initial cure assessment (a variation of approximately 6 weeks) for a disease that evolves in months is fully justifiable and, according to our understanding, would not prevent a quantitative assessment. We understand that the term "arbitrarily" used to support this choice was not properly used, and the text was reformulated (line 139) to reflect this correction. In turn, we completely agree that there is enormous heterogeneity that limits the interpretation of summary effect measures. However, the exercise of explaining differences and comparing multiple analyzes including and excluding studies based on their methodological characteristics is recognized today as a very useful strategy for understanding imperfect evidence. In this sense, the proposal is not to make statements based on summarized measurements, but to discuss directions of effects based on a critical and thorough analysis of the critical methodological aspects of each study. This concept was strongly reinforced.

**2. The results presented are clear and in line, again, with reporting guidelines. Authors have presented their findings in a very clear and succinct form. However, in line with the comment on the methods, the pooled estimates of cure rates with AmB treatment are difficult to interpret. The clinical studies included were conducted in populations infected with different Leishmania species, particularly OWCL and American species, which are known to differ in the rates of self-healing and response to treatments.**

*Authors’ comment:* We appreciate your suggestion. In addition to the topical amphotericin B combined cure rate, we also present measures stratified according to the location of the study. This information can now be seen in Figure 2 (lines 256-265).

**2.1 Regarding the presentation of adverse events, did any other of the studies (besides Lopez, 2018) assessed systemic effects (e.g., electrolytes, creatinine, transaminases, etc.)? If so, I would recommend stating in the manuscript whether systemic effects were assessed in these clinical studies and if none of them observed, which is different from lack of collection of systemic effects (e.g., no systematic measurement of blood chemistry and other markers).**

*Authors’ comment:* Systemic adverse events were exclusively reported in the study conducted by Lopes et al., 2018. Notably, the study by Goyonlo et al., 2014 mentioned the assessment of systemic allergic reactions in patients treated with intralesional amphotericin B (2 mg/mL); however, no such reaction was observed. This information has been incorporated into the text in lines 277-283.

**2.2 In addition, it would be informative to describe the duration of these adverse events, particularly those related to intralesional administration. If not available, at least would be interesting to know if some of these are permanent. For example, table 4 describes prolonged pain (n=17/93) and fibrosis (n=12/93) at the injection site (Goyonlo, 2014) in about 13-18% of treated cases, which would be important parameters to consider the feasibility of conducting further evaluations of intralesional AmB, given their potential impact in patient preference.**

*Authors’ comment:* In the study by Goyonlo et al., 2014, the authors documented the occurrence of pain during injection, which persisted for a period longer than 30 minutes. Conversely, Goswami et al., 2019 reported that pain during injection did not extend 30 minutes. It is crucial to emphasize that in both studies, the observed adverse effects did not prompt the premature discontinuation of treatment. This suggests that these adverse events, although present, were not deemed impactful enough to render the treatment unfeasible and other studies with this intervention. Comprehensive information regarding the duration of adverse events available in the studies has been presented in Table 4 and further clarified in the section addressing adverse events, particularly in lines 268-283.

**3. Abstract: The conclusion stated in the abstract ('Amp.B is a promising strategy") is not well supported. Given that the abstract is a standalone section, I would suggest topresent more details in the results of the abstract (e.g., safety or other clinical outcome) that supports the 'promising' nature of the strategy (or use other qualifications to describe the intervention).**

*Authors’ comment:* Thank you for your suggestion, the summary was reworded (lines 13-36)

**3.1 Main text: The discussion section is clear and many of the limitations of the available literature are presented. Less is discussed about the decisions made by the author team and the implications on result interpretation, for example, the decision to ‘assume’ that evaluations were at D90 in the studies, and mostly, the decision of continuing with a meta-analysis given the high heterogeneity of the studies.**

**Authors’ comment:** Thank you for your useful comment. Our methodological choices were revised and justified. Evaluating the results using a quantitative approach through meta-analysis aimed to explore the heterogeneity. Again, we reviewed the manuscript to ensure that the limitation of the summary measure was clearly stated while the focus on the analysis of the causes of the differences was highlighted.

**3.2 In the pre-clinical studies, were there any PK or toxicity studies retrieved with the search strategy? All the included one’s present outcomes related to cure, so, it would be good to know if the lack of PK/toxicity data was an artifact of the strategy selected to conduct the search.**

*Authors’ comment:* Thank you for the opportunity to clarify this point. The primary outcome of interest in this systematic review was the cure rate achieved through the local administration of amphotericin B. Studies exclusively assessing toxicity and/or pharmacokinetic information were not included. However, it is noteworthy that some included studies also reported pharmacokinetic and toxicity data, and this information has been incorporated into lines 333-351. In general, the studies indicated that intralesional administration of deoxycholate amphotericin B exhibited a swift transition into the bloodstream, potentially influencing its efficacy, a factor that could be modified by employing poly (lactic-co-glycolic acid) (PLGA) as a carrier (Sousa-Batista et al., 2019). Furthermore, topical applications with various carriers demonstrated minimal systemic toxicity, underscoring the significance of the selected administration route (Frankenburg et al., 1988; Corware, 2011; and Alves et al., 2020).

**3.3 In terms of conclusions, little could be inferred beyond the lack of standards and limited evidence for the use of AmB. This is a finding on its own and very relevant. However, it would be difficult to conclude, as authors have made for example, that continuing with intralesional administration of commercially available AmB is the best option. As mentioned in the results, more details on the safety would also help inform such a conclusion, but this is only briefly mentioned in the manuscript.**

*Authors’ comment:* Many thanks for helping us find the most appropriate position for this piece of evidence. Our intent was to contribute meaningfully to the identification of the most feasible therapeutic strategies for further development and prioritization in future research projects and not to suggest the incorporation of these results into the clinical practice. In this sense, the suggestion of amphotericin B as a potentially eligible intervention to be included in a development plan is not based purely on efficacy data but mainly on other aspects related to its commercial availability - which would allow a short-term use and its relatively well-known systemic toxicity profile. Additionally, the evidence that is progressively accumulating regarding the intralesional infiltration of antimony in the treatment of cutaneous leishmaniasis further supports this rational. All these aspects have been included in the discussion (lines 413-422).

**4. The figures are barely legible. This may have been an effect during the creation of the pdf for review, but fig 2 and 2 are almost impossible to read (very small font size and blurred text).**

*Authors’ comment:* Thank you for your observation. We checked the Plos Neglected Tropical Diseases author guidelines, and all necessary modifications were made to meet publication requirements.

**5. The study seeks to gather evidence in a very important topic, for which the number and quality of available published articles is limited. This is reflected in the results and discussion of the current systematic review. The main concern is the decision of authors to push for a meta-analysis in this context, which makes the results very difficult to interpret and potentially misleading. For example, the pooled cure rate for topical administration of AmB, was 45.6% [CI: 27.5–64.8%; I2 = 79.6), but the larger studies contributing to this number have very low cure rates (27%) while the smallest study, mostly in L. major (n=22), reported 81% cure. This is mentioned in the discussion, but one may argue that a qualitative synthesis of the available literature would allow to preserve these nuances when describing in the evidence. Given the objective of the study, a more detailed structure in the presentation of safety data (AEs), to see whether e.g., systemic AEs did not occur vs were not collected in the study, would be benefitial. Other comments to the manuscript are detailed in the respective sections.**

*Authors’ comment:* We appreciated your suggestions. They were meticulously attended to throughout the manuscript.
