# Supplementary material for: Local amphotericin B therapy for Cutaneous Leishmaniasis: A systematic review
Source: PLoS Negl Trop Dis. 2024 Apr 16;18(4):e0012127. doi: 10.1371/journal.pntd.0012127 (PMC11051593; doi:10.1371/journal.pntd.0012127)
Supplement: Attachment — Submitted filename: Response to Review_R2.docx [file pntd.0012127.s007.docx]

March 01, 2024

Dear Dra. Ana Laura Grossi de Oliveira,

We are grateful to the reviewers for their time and valuable contributions to improve the manuscript **"Local amphotericin B therapy for cutaneous leishmaniasis: a systematic review"** (**PNTD-D-23-00751R1**). We have carefully considered all reviewers’ concerns and did our best to improve the text accordingly. In all cases we’ve included in the manuscript the required changes. The modifications implemented in this revised version are summarized below together with specific answers to the questions placed by the reviewers.

Please let us know in case any other improvement in the manuscript becomes necessary.

Sincerely,

Líndicy Leidicy Alves

lindicy.leidicy@hotmail.com

**Reviewer #2:**

1. **. Regarding adverse events (table 4 and related texts), it is still not clear whether systemic adverse events were not inquired (i.e., no blood test or questions were performed as part of the study) in some of the studies instead of not reported. This distinction is very important to understand the systemic effects of the intralesional or topical administration of amphotericin B.**

*Authors’ comment:* Thank you for the opportunity to better clarify this point. The systemic adverse event was evaluated only by Lopez et al., 2018. In the other included studies, no systemic adverse effect was reported. This information was incorporated in the 274-275 lines.

**2. The discussion has improved, and the limitations of the data analyzed are better presented. Some additional comments include:**

**Lines 340 - 341: please add the confidence intervals when describing the pooled estimate of the clinical studies.**

**Lines 349 - 351: please add references to this statement.**

**Lines 372 - 373: Please verify the grammar of this sentence, it is not clear what is the meaning of: "It is and the comparison of studies."**

**Lines 384 – 390: Authors indicate that there is "lack of association" between the concentration and frequency of administration with the outcome. However, most of the studies are small and it is difficult to discern whether the lack of observed effect is real or due to the small sample size. In this case one can argue that there is no evidence of an association (due to the size of the studies), rather than a lack of association.**

*Authors’ comment:*

Lines 340-341: Confidence intervals were triggered in the text.

Lines 349-351: References have been added to the text.

Lines 372-373: The sentence was removed.

Lines 384-390: The limitations observed in these studies were highlighted previously and also in this sentence. We agree that those are small studies and it that the correct is to state the lack of evidence of association rather the lack of association. The sentence was modified to make it clearer.

**3.**  **Figures are slightly better, however, figures 2-4 are still blurred in the pdf available for review and the font size is too small. Please revise the quality of the figures (not just that they meet a file format suggested by the journal, but that the font size and layout are legible). Some of the tables have missing horizontal lines (Table 2 and 4).**

*Authors’ comment:* Thank you for your suggestion. We managed to increase the font size a little, but unfortunately, the current image is at the maximum possible resolution provided by the software. The quality of these images is much higher than the required by the Plos and we think it allows the reader to understand them well.

**Reviewer #3:**

**1. Leishmaniasis is still a disease epidemic in many countries, and effective therapeutic and preventive tools are still missing but urgently needed. In this manuscript the authors systematically reviewed the efficacy of treatment approaches with two AmB-based regimes on CL. The data were gathered mainly from studies carried in Iran, Colombia and India in the last 12 years. The cure rates significantly varied with very low certainty, which were likely determined by various factors including parasite species, subject physiological conditions and others. The review is comprehensive, but the significance of the study may be limited due to the lack of novelty of the treatment regimes.**

*Authors’ comment:* We appreciate your comment and understand your point of view, however, the main objective was to map the current level of evidence, providing the basis for a new plan for developing a therapeutic approach and designing future clinical trials.
